# Quantifying Wetting Dynamics with Triboelectrification

**DOI:** 10.1002/advs.202200822

**Published:** 2022-06-08

**Authors:** Xiaolong Zhang, Michele Scaraggi, Youbin Zheng, Xiaojuan Li, Yang Wu, Daoai Wang, Daniele Dini, Feng Zhou

**Affiliations:** ^1^ State Key Laboratory of Solid Lubrication Lanzhou Institute of Chemical Physics Chinese Academy of Sciences Lanzhou 730000 China; ^2^ Hubei Key Laboratory of Hydroelectric Machinery Design & Maintenance China Three Gorges University Yichang 443002 China; ^3^ Department of Engineering for Innovation University of Salento Monteroni‐Lecce 73100 Italy; ^4^ Department of Mechanical Engineering Imperial College London South Kensington Campus London SW7 2AZ UK; ^5^ Istituto Italiano di Tecnologia (IIT) Center for Biomolecular Nanotechnologies Via Barsanti 14 Arnesano (LE) 73010 Italy; ^6^ Qingdao Center of Resource Chemistry and New Materials Qingdao Shandong 266100 PR China

**Keywords:** hierarchical topography, infiltration dynamics, super‐hydrophobicity, TENG, theory, triboelectricity, Wetting dynamics

## Abstract

Wetting is often perceived as an intrinsic surface property of materials, but determining its evolution is complicated by its complex dependence on roughness across the scales. The Wenzel (W) state, where liquids have intimate contact with the rough surfaces, and the Cassie–Baxter (CB) state, where liquids sit onto air pockets formed between asperities, are only two states among the plethora of wetting behaviors. Furthermore, transitions from the CB to the Wenzel state dictate completely different surface performance, such as anti‐contamination, anti‐icing, drag reduction etc.; however, little is known about how transition occurs during time between the several wetting modes. In this paper, wetting dynamics can be accurately quantified and tracked using solid–liquid triboelectrification. Theoretical underpinning reveals how surface micro‐/nano‐geometries regulate stability/infiltration, also demonstrating the generality of the authors’ theoretical approach in understanding wetting transitions. It can clarify the functioning behavior of materials in real environment.

## Graphical abstract

Transitions between wetting state dictate surface performance, such as anti‐contamination, anti‐icing, drag reduction etc.; however, little is known about how transition occurs between the several wetting modes. In this paper, wetting dynamics can be accurately quantified using solid–liquid triboelectrification. Theoretical underpinning reveals how surface micro‐/nano‐geometries regulate stability/infiltration, also demonstrating the generality of the authors’ approach in understanding wetting transitions.

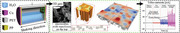

## 1 Introduction

Materials with tailored wettability have attracted much attention due to the very high demand for this feature in our daily activities and industrial applications. In particular, superhydrophobic materials, originally inspired by the performance of lotus leaves and other natural systems, have become an important scientific focus in the past thirty years.^[^ ^1^, ^2^, ^3^ ^]^ Due to the existence of a stable “air mattress” between water and superhydrophobic materials, such surface typically shows a high water contact angle (>150°) and low hysteresis angle (less than 5°). The air mattress acts as an intermediate layer supporting the droplets and decreasing its adherence, via the Cassie–Baxter state,^[^ ^4^ ^]^ hence conferring the superhydrophobic materials excellent antifouling, ant‐icing, self‐cleaning, and drag reduction properties.^[^ ^5^ ^]^ However, the non‐wetting CB state is typically metastable, with many factors, such as vibration, evaporation, air diffusion, and impact^[^ ^6^ ^]^ being able to drive the wetting transition from the CB to the Wenzel state; this may lead to significant negative outcomes, including the increase of flow resistance and ice adhesion, the aggregation of marine fouling organisms and contaminants,^[^ ^7^ ^]^ and an increased blood or bacteria adhesion. Therefore, a real‐time and facile monitoring of surface wettability to predict actual substrate performance as well as its behavior throughout products’ lifetime would be a major breakthrough in the technology of materials with designed wettability. Several studies have been devoted to the understanding and in situ monitoring of the wetting transition.^[^ ^8^, ^9^, ^10^, ^11^, ^12^ ^]^ Duan and co‐workers detected surface wettability transition from the CB to the Wenzel state using confocal microscopy.^[^ ^6^ ^]^ Contact angle and contact angle hysteresis measurements are also typically adopted (including in this work) to qualitatively observe the occurrence of wetting transitions. Nevertheless, such techniques require the adoption of laboratory equipment unlikely to be portable throughout the whole application lifetime. In this respect, being able to directly quantify the wetting dynamics and related wetting transitions by inferring the wetting state through the direct measurement of a physical marker—intrinsically linked to wetting transitions—would be greatly useful for understanding materials functioning performance.

**Figure 1:**
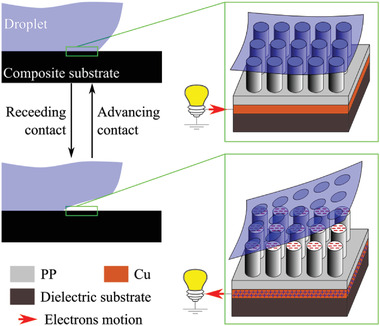
Schematic representation of tribocharging and triboelectricity processes, for a generically rough dielectric (polypropylene [PP] in the figure, with copper backplane) in multiple wetting/dewetting contacts with a water droplet. The total amount of tribocharges, thus the tribocurrent, is linearly proportional to the dewetted area during the generic contact cycle.

We note that the solid–liquid triboelectric effect is a type of contact electrification in which certain solids become electrically charged after they come into contact and separate from liquids.^[^ ^13^, ^14^, ^15^, ^16^, ^17^ ^]^ It is highly dependent on different interfacial characteristics,^[^ ^18^ ^],^ that is, surface functionalization and micro‐/nano‐structuring,^[^ ^19^, ^20^, ^21^, ^22^, ^23^, ^24^, ^25^ ^]^ and strongly linked to the wettability of the solid surfaces, as shown in the schematic representation of **Figure** **1**. Transient triboelectricity (approximately‐rectangular nano‐ to micro‐current) results from screening the triboelectric (surface) charges onto a conductive backplate; however, periodic loading/unloading contacts are needed to generate wave currents. This can happen through water sloshing or intermittent flow caused by, for example, rain or wave impacting triboelectric active surfaces, which is the current focus of blue energy harvesting research^.[^ ^26^, ^27^ ^]^

Differently from the solid–solid contact mechanics and related physics of tribocharge generation, for liquid‐based triboelectric nanogenerators (TENGs) no interface elastic energy is stored at the interface during contact, letting the wetting/dewetting dynamics and the total surface charge formation^[^ ^18^, ^27^, ^28^ ^]^ to be regulated only by surface energies. In particular, the detailed roughness across the length scales of the active surface dictates the formation of multiple stable, metastable, and unstable liquid–solid contact configurations, depending on the history of fluid squeezing pressure as well as on other environmental conditions.^[^ ^29^, ^30^, ^31^, ^32^ ^]^ Therefore, considering that the amount of true wetting area is intimately linked to the wetting/dewetting and charge generation dynamics,^[^ ^33^ ^]^ thanks to the measurement of the triboelectricity, as we will show below, the dynamic evolution of the wetting state can be quickly detected and reliably quantified, which is otherwise almost impossible to realize.

The research reported in this paper not only sheds light on the mechanisms regulating the intrinsic coupling of triboelectrification and wettability in textured polypropylene surfaces, which can have direct use in the optimization of existing and the development of new TENGs devices, but it also opens a new line of research aimed at exploiting the quantification of such links to quantify dynamic transitions in wetting states through the use of well‐calibrated triboelectric systems.

## 2 Results and Discussion

### 2.1 Micro/Nanostructured Surfaces

**Figure 2:**
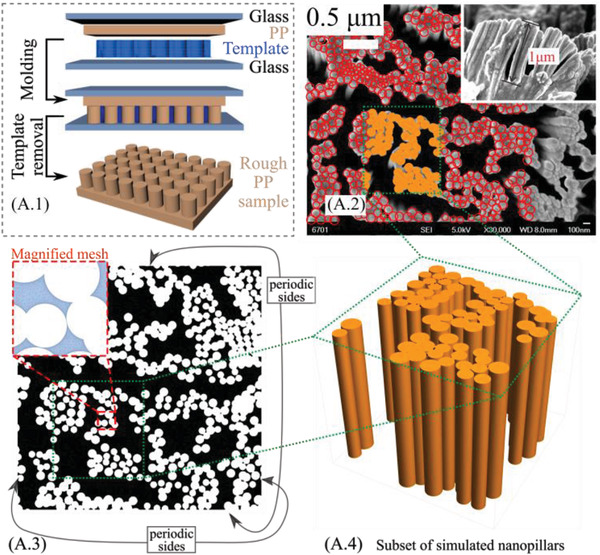
Real and virtual prototypes. The different surface roughness obtained on PP substrate after the hot molding process (A.1), including FESEM images of the nanotextured PP (A.2), with corresponding virtual prototypes (A.3 and A.4). All the virtual prototypes have periodicity along the *x*‐ and *y*‐direction.

**Figure 3:**
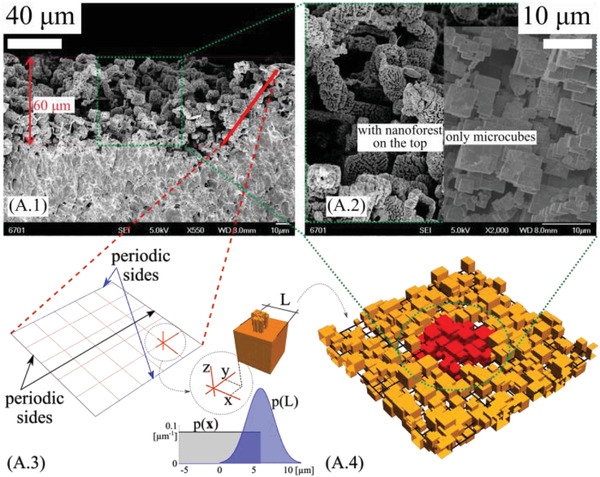
Real and virtual prototypes. FESEM image of the random distribution of microcubes (A.1) observed at length scale ≈100 µm, and magnified acquisitions of the (A.2‐right) microtextured PP and (A.2‐left) hierarchical PP, with corresponding virtual prototypes (A.3 and A.4). All the virtual prototypes have periodicity along the *x*‐ and *y*‐direction.

Polypropylene (PP) is selected as the solid of the triboelectric pair, mainly for its low surface energy/high electronegativity and widespread usage in biomedical applications and microfluidics. In our experiments, four typical samples are prototyped, that is, smooth PP, and PP with random micro‐, nano‐, and hierarchical surface topography, easily replicated by hot press molding (see the schematic of the fabrication process in **Figure** **2**A.1 and the Experimental Section for the templates preparation; the field emission scanning electron microscopy (FESEM) images of the nano‐, micro‐, and hierarchical texture are reported, for different magnifications, in Figures S2B, S2C, and S2D, Supporting Information, respectively). We observe that the choice of random surface textures, instead of deterministically designed textures with predetermined geometries, is due to the simplicity and versatility of molds preparation. The latter requires a maskless lithography process, thus no specific lithography equipment availability and surface size and planarity limitations. This makes them suitable candidates for the facile fabrication of surfaces with tailored and detectable wetting properties. As shown in Figure 2A.2, the nano‐structured PP tribo‐layer is characterized by a forest of nanowires (mean diameter ≈100 nm, Figure S2B.3, Supporting Information) with similar height (≈1 µm), arranged in randomly distributed clusters as also shown in the virtual prototypes (Figure 2A.3,A.4). The hierarchical substrate (**Figure** **3**A.1) is characterized by the superposition of the aforementioned nano‐pillar forest on the top of a pattern of randomly distributed micro‐cubes, see the magnified FESEM image in Figure 3A.2‐left. Furthermore, Figure 3A.2‐right shows the micro‐structured substrate (without the nanopillars on the top). The PP microcubes have a mean side of ≈6 µm.

A strong triboelectric signal requires a large amount of reversible surface tribocharges formation, as would be the case when adopting rough surfaces to increase the true interaction area under reversible droplet approach/separation kinematics.^[^ ^34^, ^35^, ^36^, ^37^, ^38^ ^]^ However, reversibility is highly dependent on the history of wetting pressures of the liquid–surface interactions, thus the triboelectric signal is expected to be strongly dependent on the actual wetting state. To develop models that capture the physical processes linking wetting and tribocharging dynamics, the statistical properties of the fabricated surfaces were extracted from the FESEM images such as reported in Figures 2 and 3.

The analysis of a number of surfaces allowed to derive the probability distribution functions (PDFs, Figure 3A.3) statistically representative of size and position of the micro‐blocks, as well as of the nanopillars (Figure S3A.1, Supporting Information), which were used to generate virtual prototypes (e.g., Figures 2A.4 and 3A.4) with the same statistical content of the fabricated surfaces. Note that a relatively large separation of length scales exists between the macroscale system and the microscale texture, as well as between the microscale texture and nanoscale topographic features, which is invoked as we build our multiscale wetting model.

### 2.2 Effective Wetting Properties through Simulations

**Figure 4:**
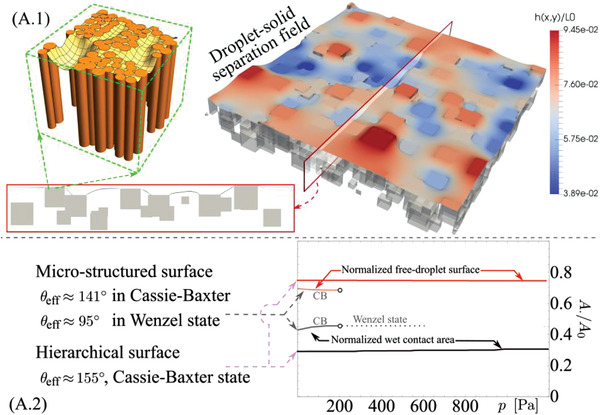
Theoretical results. A.1) Wet contact mechanics from simulations for the hierarchical surface, A.2) effective contact angle and contact area as a function of the squeezing pressure for the hierarchical and micro‐textured surface.

The contact scenarios between PP and water were simulated by a multiscale approach using the virtual prototypes described in the previous section. The initial step is to derive the surface free energy for a droplet deposited on the different PP surfaces and to study the link between fluid squeezing pressure and wetting states as discussed in Sections S1–S3, Supporting Information. Starting from the nano‐textured surfaces, it is shown that the pressure needed for a complete transition from Cassie–Baxter to Wenzel state is relatively large (≈MPa) and unlikely to be obtained for the typical operative conditions of the systems under consideration here, leading to a stable droplet true contact area, such as shown in Figure S3A.3‐top, Supporting Information. The calculated effective contact angle for the sessile droplet is *θ*_N_ ≈ 123°. Simulation of micro‐textured surfaces in the absence of superimposed nano‐texture is done with the numerical model reported in Section S3, Supporting Information, using the virtual prototypes as described in Figure 3A.4. The droplet‐solid separation field can then be computed (see Figure S3B, Supporting Information, and zoomed‐in details) and the wetted and free‐droplet zones can be determined as a function of the applied fluid pressure, as shown in Figure S3B.3, Supporting Information. At an average squeezing pressure larger than ≈200 Pa, an abrupt variation of wetting state occurs (from CB to W), which coincides with a breakdown of the stability of the numerical model. Thanks to the development of the multiscale wetting formulation (Section S1, Supporting Information), the application of the nano‐pillar structures on top of the micro‐textured PP structures (**Figure** **4**A.1) is shown to induce a drastic change of the wetting behavior. The predicted effective contact angle grows drastically with respect to the micro‐ and nano‐textured patterns, and a stable CB state for all pressures relevant to this investigation is found, as shown in Figure 4A.2. The hierarchical surface thus behaves as a robust CB‐surface, whilst increasing the true contact area with respect to the flat smooth PP thanks to the larger surface area introduced by the micro‐texture, see Figure 3A.1.

**Figure 5:**
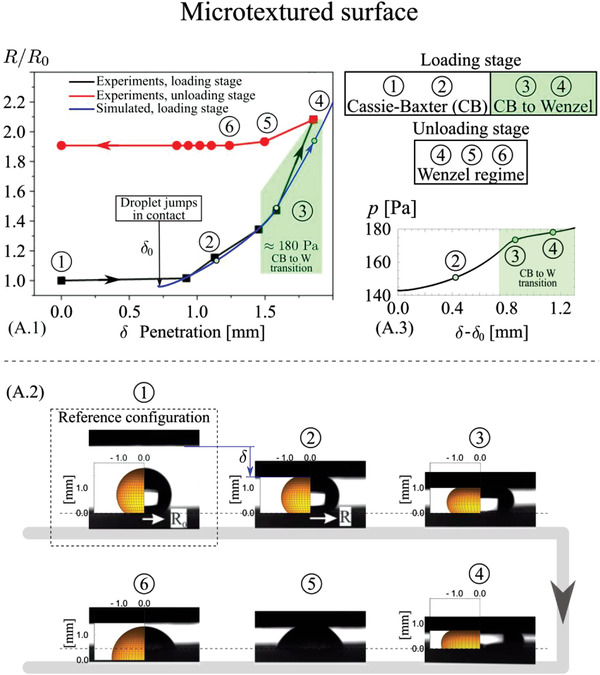
Experimental and simulation results of macroscopic dynamic wetting properties, under quasi‐static kinematics, for the microtextured surface. In the droplet simulations, the effective contact angle as a function of the droplet relative pressure is taken from the simulations results of Figure 4 dynamic (quasi‐static) wetting properties for the microtextured surface. A.1) Measured relative contact radius *R*/*R*_0_ as a function of the penetration during loading ((1) to (4), black curve) and unloading ((4) to (6), red curve) stages. The blue curve (only loading stage) is the theoretical prediction, whereas the green area represents the range of pressure and penetration at which the Cassie–Baxter to Wenzel transition occurs. (A.2) shows the comparison between the predicted and measured droplet shapes at the varying squeezing pressure. (A.3) shows the calculated contact pressure as a function of the penetration. Note the neat curve bending occurring during the CB to W transition, which occurs for a range of water squeezing pressure of ≈180 Pa.

**Figure 6:**
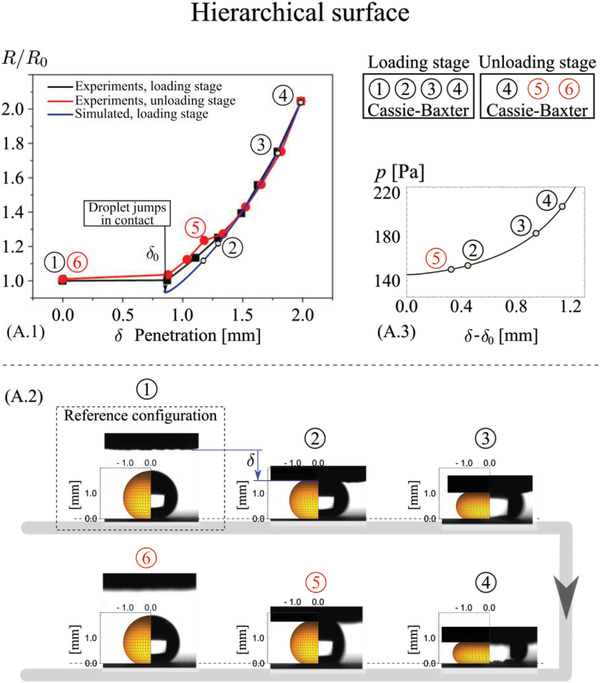
Experimental and simulation results of macroscopic static and dynamic wetting properties, under quasi‐static kinematics, for the hierarchical surface. In the droplet simulations, the effective contact angle as a function of the droplet relative pressure is taken from the simulations results of Figure 4 dynamic (quasi‐static) wetting properties for the hierarchical surface. A.1) Measured relative contact radius *R*/*R*_0_ as a function of the penetration during loading ((1) to (4), black curve) and unloading ((4) to (6), red curve) stage. The blue curve (loading/unloading stage) is the theoretical prediction, characterized by a stable Cassie–Baxter regime. (A.2) shows the comparison between the predicted and measured droplet shapes at the varying squeezing pressure. (A.3) shows the calculated contact pressure as a function of the penetration. *R*_0_ is the droplet radius in the sessile contact configuration. Note that the differences between (A.3) and Figure 5A.3, in particular for the hierarchical case the curve slope is monotonically increasing with the penetration.

A comparison between the wetting properties predicted by the theory (apparent contact angle, contact radius, and contact force) and the results obtained experimentally are given in Figure S5, Supporting Information, for the sessile‐droplet experiments, and in **Figures** **5** and **6** for the dynamic squeezing‐droplet experiments. For all the shown droplets, the left half represents the simulated and right half the real one. It shows the capability of our models to capture the effective or apparent contact angle, thus the true wet contact area, for all investigated surfaces. The dynamic wetting behavior of the four surfaces was investigated since the energy exchanged at the triboelectrification interface, thus the triboelectric signal, is primarily determined by the contact mode at the liquid–solid–gas interface and its evolution at varying droplet squeezing pressures. The sessile droplet was thus pressed and released with a superhydrophobic top‐plate (Figures 5 and 6) to shed light on the wetting transition under external pressure, which corresponds to changes of the apparent radius of contact, *R*, with respect to the initial (sessile) contact radius, *R*_0_. Macroscopically, the droplet is shown to undergo different transitions depending on the texture of the PP surface under consideration. For the micro‐textured PP surface, the droplet does not recover its initial shape after release but shows a much lower contact angle due to the transition from Cassie‐Baxter to Wenzel state during the compression stage. Data in Figure 5A.1,A.2 shows a marked difference between loading and unloading curves, with a clear transition at ≈180 Pa, consistent with the numerical predictions. Interestingly, after the transition to Wenzel state is completed, part of the droplet mass content is trapped in the micro‐structured surface, as suggested by the comparison with the predicted sessile droplet shape. On the other hand, the hierarchical PP surface (Figure 6) shows a remarkably stable CB state. The agreement with the theory in term of droplet pressure versus displacement curves and apparent contact angles does validate the solid–liquid true contact area theoretical predictions.

Quantitative measurements of the residual droplet volume with force measurements are given in Figure S8, Supporting Information.

### 2.3 Quantitative Tracing of Wetting Dynamics by Triboelectrification

**Figure 7:**
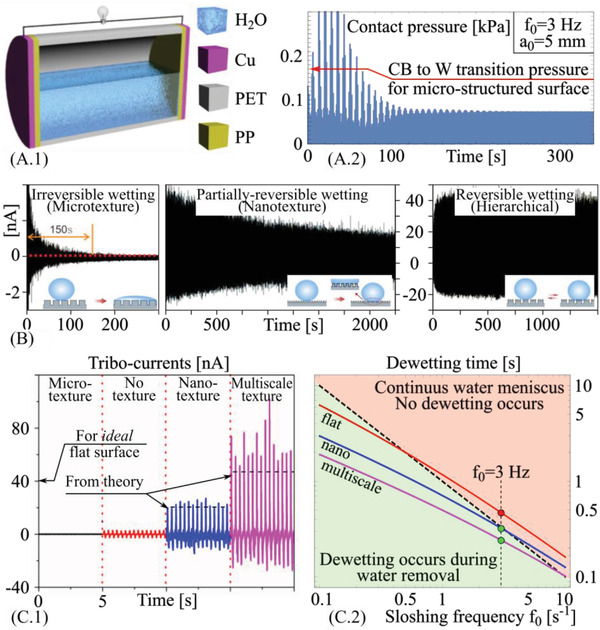
Theoretical and experimental results. A.1) Our TENG device, A.2) typical TENG sloshing pressure, and B) tribocurrent as a function of time. C.1) Steady‐state tribocurrents and C.2) drag‐out dewetting map.

To experimentally investigate the link between triboelectric signal and dynamic wetting, the PP substrates were integrated onto both ends of a cylindrical tank (schematic in **Figure** **7**A.1), subjected to vibration along the cylinder axis via a linear actuator at varying frequencies. The tank displacement is set sinusoidal, with an amplitude of 5 mm. For this prototype macro‐geometry, the triboelectrification performance is dictated by the wetting and drag‐out dewetting behavior of the surfaces, as well as by the sloshing dynamics associated with the mechanical vibration (Figure 7A.2 and Figure S10, Supporting Information). The generation of tribo‐charges as a function of time for all the structured surfaces developed here is reported in Figure 7B for the sloshing frequency of 3 Hz, whereas the steady‐state tribo‐current is reported in Figure 7C.1 and compared with the theoretical predictions (see Section S6, Supporting Information). The smooth PP film showed relatively small tribocurrent outputs, with a tribo‐charge of 0.095 nC (open‐circuit voltage *V*_oc_ of 0.12 V). The PP with micro‐textures showed negligible tribocurrent, with a tribo‐charge of ≈0.0029 nC (*V*_oc_ of 0.0067 V). For the PP with nano‐structured surface, the tribo‐charge increased to 0.51 nC (*V*_oc_ of 1.24 V). The hierarchically structured surface produced the highest output with a tribo‐charge of ≈0.94 nC (*V*_oc_ of 2.73 V, see Section S7, Supporting Information, for more details).

The link between wetting state and tribocurrent dynamics can be fully theoretically explained by investigating the individual phases of the process, starting from the sloshing dynamics induced by the vibration of the water‐based TENG constructed here; the key parameters and a schematic representation of the process are reported in Figure S10, Supporting Information. The weakly‐nonlinear sloshing dynamics model of the partially filled tank (developed as reported in Section S8, Supporting Information) enables to predict the moving free surface as well as the center of mass trajectory of the sloshing water for the conditions under consideration and as a function of the filling ratio, as shown in Figure S10A.2, Supporting Information. Capturing the dynamics of the system also allows us to determine the average fluid pressure exerted at the wall, and its relation with the shaking frequency; the results reported in Figure 7A.2 for example demonstrate how during the transient stage of the sloshing dynamics, the contact pressure exerted on the water surface increases to values which determine the transition from CB to Wenzel wetting state for the micro‐textured surface. Thus, for this case, independently of the drag out dynamics, a film of water is cumulatively entrapped onto the PP surface leading to no triboelectric effect, in perfect agreement with the experiments Figure 7C.1. However, when the sloshing frequency is reduced, the maximum squeezing pressure encountered during the shaking dynamics decreases to values leading to an incomplete Wenzel transition in the micro‐structured PP sample. This is in turn reflected in the measured tribocurrent (see Figure S12, Supporting Information), which follows the time dependence of the sloshing pressure (Figure 7A.2). In the weak non‐linear sloshing model, the initial increase in the average water pressure is due to the superposition of the steady‐state solution, driven by the excitation frequency, and the transient solution, driven by the initial excitation velocity and damped after few cycles. The average water pressure acting on the PP walls is considered in the computational of the tribocharge generation instead of the pointwise pressure function, due to the simplifying assumptions of the sloshing model.

For the smooth, nanotextured and hierarchical surfaces, the triboelectric behavior also depends on the occurrence of dynamic transitions between wetting and drag‐out dewetting as a function of the sloshing frequency. The drag‐out mechanism can be modelled by generalizing standard drag‐out theories as reported in Section S8, Supporting Information. The model can be used to describe the dependence of the time to dewet the surface on the spreading pressure and effective contact angle of the different surfaces. This can then be linked to the sloshing frequency to produce the drag‐out map reported in Figure 7C.2. It is extremely important to note that the triboelectrification is strongly affected by the synchronization between the dewetting time and the sloshing dynamics of the device. Figure 7C.2 shows how the different PP surfaces perform in terms of dewetting for different sloshing frequencies. For the 3 Hz sloshing frequency employed in the experimental set up for demonstration purposes, it is shown how the untextured surface does not reach the post‐unstable regime needed for dewetting to take place and for the triboelectric effect to manifest itself. The contrary is true for the nano‐textured and hierarchical surfaces, which indeed show transition to dewetting during the sloshing cycle and, thus, a significant triboelectric effect, whose measured values are in agreement with theory. This confirms the intimate link between actual wetting state (and the different physical mechanisms governing this) and nanocurrent signal.

The triboelectric signal originating from the micro‐textured surface is remarkably sensitive to its wetting history; in particular, the output current decreases continuously with the sloshing time (Figure S12A–C, Supporting Information), unravelling the complex wetting behavior of the microstructured PP as made by incremental irreversible local Wenzel transitions. With the increase of the vibration frequency, the time needed for the output to reach the final current also decreases with the different frequencies, from ≈3000 s at 1 Hz to ≈150 s at 3 Hz, due to the increase of the squeezing pressure with the sloshing frequency square. On the other side, for hierarchically structured PP (shown for 3 Hz in Figure S12D, Supporting Information) the tribocurrent is constant, thus the wetted area is unaffected by the variation in squeezing pressure (Figure 7A.2), also in agreement with theory.

The theoretical work presented here has enabled us to provide a direct link between the sloshing dynamics and the water pressure building at the interface of the liquid–solid TENG used for the experimental investigation presented in this work. The link between the water pressure applied at the macroscale and the transition between wetting states is indeed the key to unravel the connection between wetting dynamics and triboelectricity; this is confirmed by the fact that the wetting transition responsible for the hysteretic behavior of the droplet squeezed between textured plates, explored in Figures 5 and 6, can also be directly related to the pressure generated on the liquid film and the wetting transition observed using a sloshing device. This newly gained theoretical understanding has enabled us to link the changes in wettability of the surfaces and their possible reversibility to the physics of the problem and the triboelectric response of the device adopted for this investigation, providing results that are easily transferrable to other scenarios.

It should be highlighted here that the focus of this contribution has been on exploring the effect of texture and surface topography on wetting dynamics. The other important aspect that must be considered to obtain a fuller picture is the effect of surface chemistry and, more specifically, the consequences that chemical changes have on the surface energy and the intrinsic wetting of the surfaces. Although the exploration of this aspect is outside the scope of this work, here we note that the stability of the tribocurrent generation on the hierarchical surface does support the conclusion that the molecular mechanisms allowing triboelectric charging are reversibly occurring on the generic PP surface. However, the robustness of the superhydrophobicity on hierarchical PP can be easily broken, for example by using surfactant solutions, as discussed in detail in Section S10, Supporting Information. The presence of surfactant contaminant affects the surface tension of water, which in turn changes the interfacial energy and the equilibrium wetting area. Thus, at increasing surfactant concentration the transition between CB and Wenzel states is favored during the sloshing dynamics, as can be easily detected by the change in the electric signal reported in Figure S13, Supporting Information. The higher the surfactant concentration, the faster the decay and the smaller the equilibrium tribocurrent. This confirms the remarkable sensitivity of triboelectricity in the real‐time wetting detection and shows the promise that our methodology has in potentially linking chemical changes in both liquid and solid phases to dynamic wetting transitions.

### 2.4 Fluorescence Verification

We have employed large field imaging to investigate the link between transient behavior of triboelectricity and fluid infiltration dynamics for the different textured samples, see Figure S15, Supporting Information. These observations show that the hierarchical surface displays negligible Wenzel transitions upon multiple sloshing cycles, thus no infiltrated water can be detected on the combined micro/nano‐textured surface, as expected theoretically and demonstrated by the intrinsically linked stable triboelectric signal (Figure S12D, Supporting Information). On the other hand, the nano‐textured PP shows a uniform (due to the sampling resolution of the optical acquisition, which cannot resolve the smallest scales) reduced infiltration after 30 min sloshing time.

This definitely confirms that Wenzel transitions might occur on the nanopillar forest, but without interfering with the dynamics of tribocurrent generation. This agrees with the theoretical study reported in Figure S3A, Supporting Information, where we show that Wenzel transitions can occur for the nano‐textured surface; however, this does not lead to a substantial variation of the amount of wet area responsible for the triboelectric signal, as this corresponds to the cumulative contribution of all the pillars’ top surfaces.

**Figure 8:**
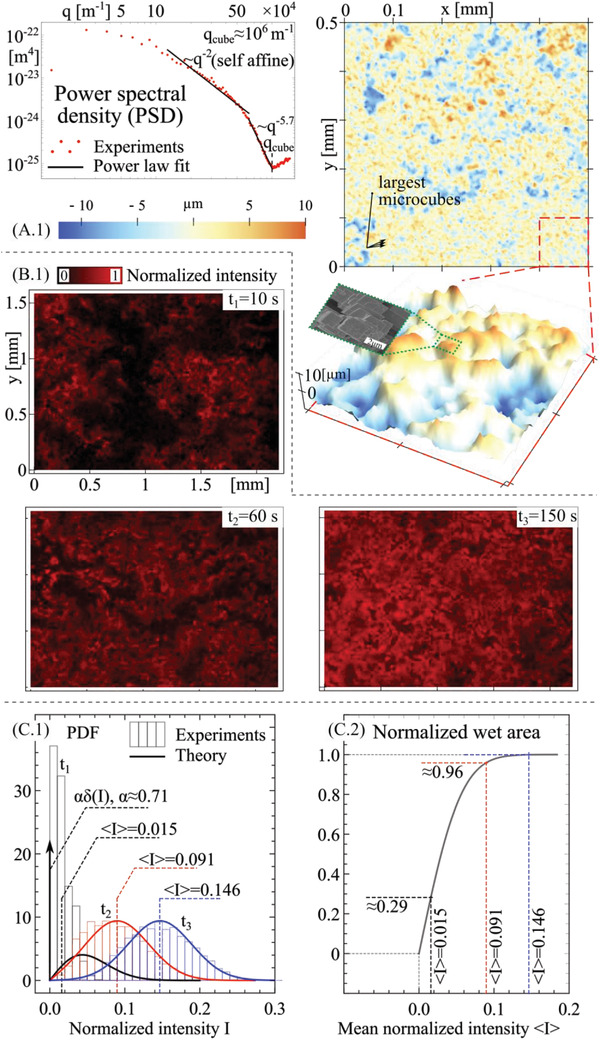
Experimental and simulation results. A.1) Space‐of‐lengths and spectral description of the microstructured surface. B.1) Epifluorescence images of the doped water infiltrated in the microtextured surfaces upon multiple sloshing cycles. C.1) Probability density function of the doped water infiltrated thickness field from experiments and theory (*F*(*q*_1_) is set to 9E‐4 to fit data). C.2) Evolution of wetted area as a function of the mean infiltrated water volume.

As expected, the most interesting infiltration dynamics occurs for the micro‐textured surface. In **Figure** **8**A.1 the topography and correlation details of the micro‐texture surface are reported from a contact stylus acquisition of the surface roughness, shown in magnified view. The largest microcubes edges are clearly visible in terms of highly fragmented small red features in the roughness map. The roughness power spectral density is also reported, with indication of a self‐affine domain (with fractal dimension ≈3, related to the random distribution of the microcubes on the surface—see Figure 3A.4) and of the mean microcube frequency *q*_cube_ ≈ 10^6^ m^–1^, related to an average microcube size 2*π*/*q*_cube_ ≈ 6.3 µm.

In the panel below, Figure 8B.1, the epifluorescence images of the doped water infiltrating the micro‐textured surface after multiple sloshing cycles are reported. The black random patterns show, interestingly, that an incomplete irreversible wetting occurs at the water/microtextured PP interface, with the dry domain decreasing at increasing sloshing times. In Figure 8C.1 the PDF of the fluorescence intensity are reported against the theoretically predicted PDFs (see Section S11 and Equation (S29), Supporting Information), at varying sloshing times. In the theoretical results, we have assumed the fluorescence intensity to be linearly proportional to the wetting film thickness, as applicable in our case. At time *t*_1_, the probability is mostly associated to the Dirac's delta function *δ*(I) with prefactor ≈0.71 (coarse‐grained in the experimental results due to the optics resolution), whereas at time *t*_3_ the PDF is mostly Gaussian, in very good agreement with experiments. The theory predicts a PDF of wetting film thickness constituted by the summation of two mirrored Gaussian distributions and a Dirac's delta function (the latter characterizing the probability associated to the non‐wetted domains), as a result of a Fokker–Planck equation describing the random film breakdown dynamics, see Section S11, Supporting Information. In Figure 8C.2 the predicted normalized projected wet area is reported as a function of the average fluorescence intensity (proportional to the average infiltrated water thickness). At time *t*_3_ most of the micro‐textured surface is wet, with nearly Gaussian distribution of the film thickness, which is again in agreement with the experiments. The irreversibly increasing water infiltration due to a progressive Wenzel transition on the micro‐textured surface thus determines the decaying of the triboelectric signal at increasing sloshing times, as clearly shown in Figure S12C, Supporting Information, for the 3 Hz case. Remarkably, this demonstrates how triboelectricity can be effectively used for the in situ monitoring of the most general wetting dynamics on newly designed surfaces, with real‐time evaluation of the wet and infiltration contact area according to the theoretical model.

## 3 Conclusions

The intimate link between triboelectricity and wetting dynamics has been unraveled for (polypropylene) polymer substrates characterized by: i) smooth; ii) micro‐structures; iii) nano‐structures; and iv) hierarchical structures. A multiscale model was developed to shed light on the mechanisms linking triboelectric generation to wetting dynamics and wetting‐induced fluid infiltration. Wetting/dewetting transition from Cassie–Baxter to Wenzel mode, mechanically induced by dynamic water pressure, or from a retarded water drag‐out process, was correlated with triboelectric decay. This, for the first time, allows us to quantify a plethora of wetting dynamics processes (and related wetting area). On one extreme, the hierarchical PP surface with structured sidewalls provides a stable and large tribo‐nanocurrent with respect to untextured and nanostructured surfaces, as a consequence of either a faster drag‐out dewetting or a larger reversible true wet contact area. On the other side, incremental infiltration dynamics appears on the microtextured surface, as unraveled too by the triboelectric signal, in real‐time. Therefore, the most important outcome of this study is the fidelity of triboelectricity to study the dynamic wetting properties of surfaces. The implications of the results obtained in this work, which also paves the way for designing high‐performance liquid‐based triboelectric devices, are extremely broad as our findings and the theoretical understanding provided in this paper can be used to, for example, develop design tools and automated testing protocols and sensing technologies to optimize surfaces and tune their wetting dynamics for a plethora of applications for which obtaining and controlling superhydrophobicity is key.

## 4 Experimental Section

### Preparation of Nanoporous Alumina (AA1) Templates

Aluminum sheets were polished with sandpaper, ultrasonically cleaned in alcohol and washed with deionized water in sequence to get rid of grease. The Al sheet was then polished with an electrochemical method in perchloric acid (HClO_4_) and ethanol mixture for 10 min with a voltage of 15 V. Finally, a two‐steps anodization method was used to prepare the AAO template in 0.3 m oxalic acid (H_2_C_2_O_4_) electrolyte with a voltage of 60 V, and the first oxidation process lasted for 2 h whereas the second anodic oxidation time for 20 min. Then, after etching in 5 vol% H_3_PO_4_ solution for 10 min to enlarge the pore size, the nanoporous alumina (AA1) templates were obtained.

### Preparation of Micro‐Structures (AA2) and Hierarchical Structure Alumina (AA3) Templates

The pre‐cleaned Al foil was electrochemically etched in a 10 g L^−1^ NaCl aqueous solution at 4 V for 3 h at room temperature to obtain micro‐structures (AA2). Second, the resultant porous alumina with overhanging micro‐structures was anodized at a constant voltage of 60 V for 20 min in 0.3 m oxalic acid at the temperature of 0 °C. Finally, after etching in 5 vol% H_3_PO_4_ solution at 60 °C for 10 min to enlarge the pore size, the hierarchical structure alumina (AA3) templates were obtained. The morphology of AA3 template is given in Figure S16, Supporting Information.

### Fabrication of the Three Different Surface Roughness PP Samples

The PP surfaces were prepared by a simplified hot processing technique using AA1, AA2 and AA3 as template, as shown in Figure S2A, Supporting Information. Typically, a flat PP film with the thickness of 50 µm and a template material were squeezed in between of two glass plates, and then moved into oven at 200 °C whilst applying a squeezing load of 2 n cm^−2^. After maintaining the temperature at 200 °C for 1 h and cooling down without releasing the load, the prepared PP composite was put into a hot NaOH solution (1.0 m) at 60 °C for 3 h to remove the AAO template. Finally, after dissolving the AAO template, the PP friction layers were ultrasonically cleaned in alcohol for 20 min. The obtained structured PP surfaces are shown in Figure S2B–D, Supporting Information.

### Fabrication of Triboelectrification Device

The PP surfaces were used as the solid phase of the triboelectric pair. Copper foil tapes were first stuck onto the backside of PP film layer, and then copper wires were attached to one side of the copper tape as a lead wire. Polyethylene terephthalate (PET) cylinder was chosen as the substrate and container material, with cylinder length being 200 mm and diameter 40 mm. The PP film was fixed on both ends of the PET tube and sealed with silicone rubber to prevent water leakage. Then, distilled water with/without surfactant was inserted into the tank, acting as the liquid phase of the triboelectric pair, see Figure 7A.1.

### Characterization

The morphologies of different surface roughness PP were acquired with a FESEM (JSM‐6701F, JEOL Inc., Japan). Contact angle (CA) measurements were done with a DSA100 contact angle meter (Kruss Company, Germany) at room temperature. The average CA value was obtained by measuring the sample at five different positions for 5 µL liquid, and the images were captured with a traditional digital camera. For measurement of the triboelectric outputs of TENG, a sinusoidal reciprocating motion was applied on the tank with a commercial linear mechanical actuator (IVCL17‐56) with controllable frequency and amplitude. The open‐circuit voltage was measured by using a NI‐PCI6259 (National Instruments), while the short circuit current was measured by using an SR570 low‐noise current amplifier (Stanford Research System), and data were collected through LabVIEW base Development System (National Instruments)

## Conflict of Interest

The authors declare no conflict of interest.

## Author Contributions

F.Z. and D.W. conceived the idea and supervised the entire research. Y.Z., Y.W., and X.L. performed the experiments and completed the whole characterizations. M.S. and D.D. carried out the simulation. Y.Z. and M.S. drafted the manuscript. F.Z. and D.D. revised and finalized the manuscript. All the authors discussed the results and provided technical suggestions.

## Supporting information

Supporting Information

## Data Availability

The data that support the findings of this study are openly available in Zenodo at https://doi.org/10.5281/zenodo.5654462, reference number 5654462.
