# Supplementary material for: Quantifying Wetting Dynamics with Triboelectrification
Source: Adv Sci (Weinh). 2022 Jun 8;9(24):2200822. doi: 10.1002/advs.202200822 (PMC9405515; doi:10.1002/advs.202200822)
Supplement: Supplementary file 1 — Supporting Information [file ADVS-9-2200822-s001.pdf]

ADVANCED SCIENCE
Open Access

Supporting Information

for *Adv. Sci.*, DOI 10.1002/advs.202200822

Quantifying Wetting Dynamics with Triboelectrification

Xiaolong Zhang, Michele Scaraggi, Youbin Zheng, Xiaojuan Li, Yang Wu, Daoai Wang*, Daniele Dini* and Feng Zhou**

Supplementary Information

for

Quantifying Wetting Dynamics with Triboelectrification

This PDF file includes:

Supplementary text, from Section S1 to S11
Figs. SS1 to SS16

SI References

Supplementary Information Text

S1. Effective wetting properties for a multiscale surface

We consider a representative elementary volume of interface at magnification *n*, where one roughness length scale dominates (say the roughness length scale $\lambda = L_0/n$, where L_0 is the system size at the macroscopic scale corresponding to magnification 1). Furthermore, we consider a large separation of length scales between the magnification *n* and the others. Such portion of interface is either in a partial wetted (see Fig.SS1 top left drawing) or dewetted (see Fig.SS1, bottom left drawing) state, depending on the history of the wetting process. In Fig.SS1 we show a schematic (generic) representation of the roughness appearing at magnification *n* and *n+1*, respectively on the left and right panel. In this study we have searched for the equilibrium configurations only for the wetting state, the latter occurring during the initial advancing contact process of the fluid against the rough substrate (see Fig.SS1, top left-right drawing). However, the model below can be similarly applied also to other partial contact states (such as shown in Fig.SS1, bottom left-right drawing).

In particular, we look for the static equilibrium by minimizing the interface energy given by:

$$U = \gamma_{\mathrm{LS}}^{n} A_{0\mathrm{c}}^{n} + \gamma_{\mathrm{SV}}^{n} A_{0\mathrm{s}}^{n} + \gamma_{\mathrm{LV}} A_{\mathrm{l}},$$

where $A_{0\mathrm{c}}^{n}$ is the apparent solid-liquid contact surface, $A_{0\mathrm{s}}^{n}$ the apparent droplet-free surface and A_{l} the droplet free-surface ($A_0^n = A_{0\mathrm{c}}^{n} + A_{0\mathrm{s}}^{n}$). Above, the superscript *n* refers to the effective physical variables as observed at the actual magnification *n*. By using the definition of effective contact angle observed in the contact at the actual magnification *n*

$$\cos\theta^{n} = \frac{\gamma_{\mathrm{SV}}^{n} - \gamma_{\mathrm{LS}}^{n}}{\gamma_{\mathrm{LV}}}$$

we get

$$U = -\gamma_{\mathrm{LV}} \cos\theta^{n} A_{0\mathrm{c}}^{n} + \gamma_{\mathrm{SV}}^{n} A_0^{n} + \gamma_{\mathrm{LV}} A_{\mathrm{l}}.$$

The equation above can be minimized referring to both the partial wetted (see Fig.SS1, top left drawing) or the dewetted (see Fig.SS1, bottom left drawing) state. However, the effective wetting surface energies γ_{SV}^{n}, γ_{LS}^{n} (or γ_{SV}^{n} and $\cos\theta^{n}$) needs to be calculated from the wetting/dewetting occurring at the scale *n+1*. Thus, when magnifying the domains which appears to be in fluid contact (Fig.SS1, green small boxes on the left panel) or not in contact (Fig.SS1, red small boxes on the left panel) at scale *n*, such domains are fragmented over multiple contacts (Fig.SS1, green large boxes on the right panel) or multiple non-contacts (Fig.SS1, red large boxes on the right panel). By equating the surface energy written in the apparent interaction areas at magnification *n* and *n+1* we get

$$\gamma_{\mathrm{LS}}^{n} = \alpha_{\mathrm{LS}}^{n+1} \gamma_{\mathrm{LS}}^{n+1} + \alpha_{\mathrm{LV}}^{n+1} \gamma_{\mathrm{LV}} + \alpha_{\mathrm{SV}}^{n+1} \gamma_{\mathrm{SV}}^{n+1}$$

and

$$\gamma_{\mathrm{SV}}^{n} = \beta_{\mathrm{LS}}^{n+1} \gamma_{\mathrm{LS}}^{n+1} + \beta_{\mathrm{LV}}^{n+1} \gamma_{\mathrm{LV}} + \beta_{\mathrm{SV}}^{n+1} \gamma_{\mathrm{SV}}^{n+1},$$

where the α_i^{n+1} and β_i^{n+1} are the normalized apparent interaction areas between the phases-*i*, respectively, inside and outside the apparent droplet-*n* contact area. In the equations above we have assumed that the effective surface energies occurring, at the generic magnification *n+1*, are equal in both the partial wetted and partial dewetted state, i.e. we have implicitly considered the fluid to be subjected to the same pressure both in the wetting and dewetting areas during the generic interaction process. We observe, however, that this assumption does not affect the calculation in the initial advancing contact process analyzed in this study.

Given that $r_{\mathrm{sub}}^{n+1} = \alpha_{\mathrm{SV}}^{n+1} + \alpha_{\mathrm{LS}}^{n+1} = \beta_{\mathrm{SV}}^{n+1} + \beta_{\mathrm{LS}}^{n+1}$ is the normalized roughness surface area, one gets the effective equations

$$\gamma_{\mathrm{LS}}^{n} = \gamma_{\mathrm{LV}} (\alpha_{\mathrm{LV}}^{n+1} - \cos\theta^{n+1} \alpha_{\mathrm{LS}}^{n+1}) + r_{\mathrm{sub}}^{n+1} \gamma_{\mathrm{SV}}^{n+1} \qquad (S1)$$

and

$$\gamma_{\mathrm{SV}}^{n} = \gamma_{\mathrm{LV}} (\beta_{\mathrm{LV}}^{n+1} - \cos\theta^{n+1} \beta_{\mathrm{LS}}^{n+1}) + r_{\mathrm{sub}}^{n+1} \gamma_{\mathrm{SV}}^{n+1} \qquad (S2)$$

leading to an effective contact angle

$$\cos\theta^{n} = \cos\theta^{n+1} (\alpha_{\mathrm{LS}}^{n+1} - \beta_{\mathrm{LS}}^{n+1}) - (\alpha_{\mathrm{LV}}^{n+1} - \beta_{\mathrm{LV}}^{n+1}). \qquad (\mathrm{S3})$$

Equations (S1-S3) need to be applied recursively for the case of a multiscale roughness, as is the case in our investigation, as shown in Fig.SS2. Clearly, in order to determine the effective wetting properties at scale-*n* (γ_{LS}^{n}, γ_{SV}^{n} and $\cos\theta^{n}$), one needs not only the effective wetting properties at

scale *n+1* ($\gamma_{\mathrm{LS}}^{n+1}$, $\gamma_{\mathrm{SV}}^{n+1}$ and $\cos\theta^{n+1}$), but also the fraction of fluid surface in contact/non-contact with the substrate at magnification *n+1*. For our systems, we have three representative length scales: the macroscale of the droplet (say *n=1*), the intermediate micro-scale (given by the randomly placed micro-cubes, say magnification *n=2*) and the highest magnification at the nano-forest scale (say *n=3*). Thus, we have solved first the static contact process at the nanoscale in Sections S2, whose results are then used to minimize the interface energy in Section S3 at the microscale. Finally, the results from Section S3 are adopted to determine the effective surface energies at the macroscale droplet contact problem.

S2. Effective wetting properties for the nano-textured surface

Here we study first the effective wetting properties of a hexagonal lattice of cylindrical pillars, with a flat top surface. The pillars have radius R_{p}, height h_{p} and intra-cluster lattice size L_{p}. We assume the generic local maximum penetration of the droplet in the pillar forest, δ, to be $\delta \ll L_{\mathrm{p}}$ (i.e. local approximation of the droplet surface as paraboloid, valid for small droplet penetration compared to the hexagonal lattice size L_{p}); by applying the Young-Laplace equation, this leads to $\delta \approx \left(L_{\mathrm{p}} - 2R_{\mathrm{p}}\right)^2 p/(8\gamma_{\mathrm{LA}})$, where p is the droplet relative squeezing pressure and where we have assumed the mean droplet curvature to be $\approx 8\delta/\left(L_{\mathrm{p}} - 2R_{\mathrm{p}}\right)^2$. The maximum fluid pressure allowed to keep a Cassie-Baxter (CB) wetting regime, by geometric considerations, is the minimum between

$$\frac{p_{\max}L_{\mathrm{p}}}{\gamma_{\mathrm{LA}}} = -\cos\theta_0 \frac{\frac{8R_{\mathrm{p}}}{L_{\mathrm{p}}}}{\frac{2\sqrt{3}}{\pi} - \frac{4R_{\mathrm{p}}^2}{L_{\mathrm{p}}^2}} \qquad (S4)$$

and

$$\frac{p_{\max}L_{\mathrm{p}}}{\gamma_{\mathrm{LA}}} \approx \frac{h_{\mathrm{p}}}{R_{\mathrm{p}}} \frac{\frac{8R_{\mathrm{p}}}{L_{\mathrm{p}}}}{\left(1 - \frac{2R_{\mathrm{p}}}{L_{\mathrm{p}}}\right)^2}, \qquad (S5)$$

whereas the average penetration occurring for pressures $p_0 < p_{\max}$ is:

$$\delta_{\mathrm{p}} \approx \frac{\left(L_{\mathrm{p}} - 2R_{\mathrm{p}}\right)^2}{8} \frac{p_0}{\gamma_{\mathrm{LA}}} \left(1 - \frac{R_{\mathrm{p}}^2}{L_{\mathrm{p}}^2} \frac{2\pi}{\sqrt{3}}\right).$$

Given that $\frac{8R_{\mathrm{p}}/L_{\mathrm{p}}}{2\sqrt{3}/\pi - 4R_{\mathrm{p}}^2/L_{\mathrm{p}}^2}$ or $\frac{8R_{\mathrm{p}}/L_{\mathrm{p}}}{\left(1-2R_{\mathrm{p}}/L_{\mathrm{p}}\right)^2}$ are factors of order 1 for our system, the pressure required for the wetting transition from the CB to Wenzel (W) state will thus be related to the minimum value between the pillar aspect ratio, $h_{\mathrm{p}}R_{\mathrm{p}}^{-1}$ and the effective contact angle, $-\cos\theta_0$. For our nano-forest $2R_{\mathrm{p}} \approx 100$ nm, $h_{\mathrm{p}} \approx 1$μm. Therefore, $h_{\mathrm{p}}R_{\mathrm{p}}^{-1} \approx 20$, whereas $-\cos\theta_0 \approx 0.2$, leading to the conclusion that, for our system, (S4) is the relevant equation describing the squeezing pressure necessary for the transition CB to W to occur for a hexagonal lattice of flat pillars. Eq. (S4) shows that the minimum intra-cluster distance at which the CB to W transition occurs quickly decreases at increasing squeezing pressures. This is shown in Fig.SS3, where it is shown (A3) that a Wenzel transition would occur at intra-cluster distances of about 3 μm for a squeezing pressure of order kPa. This intra-cluster value is much larger than the average intra-cluster distance occurring in our system, see Fig.SS3(A.1 - bottom) and below.

For our system, pillars are arranged in local clusters, with inter-cluster distance probability distribution reported in Fig.SS3(A.1 - top), and intra-cluster lattice spacing probability distribution reported in Fig.SS3(A.1 - bottom). The latter is well fitted with a double Gaussian (see solid line in the above-mentioned figure)

$$P\left(L_{\mathrm{p}} > L_1\right) \approx \mathrm{a}_0 \left(e^{-\frac{1}{2}\frac{\left(L_{\mathrm{p}}-L_1\right)^2}{L_2^2}} - e^{-\frac{1}{2}\frac{\left(L_{\mathrm{p}}+L_1\right)^2}{L_2^2}}\right), 0\ elsewhere \qquad (S6)$$

where $\int dL_{\mathrm{p}}\, P\left(L_{\mathrm{p}}\right) \approx 0.57$, representing the total top surface pillars area normalized with the substrate area. The CB to W transition is dictated by the wetting occurring at the cluster scale. Thus, in order to predict the effective contact angle for the random nano-pillar forest, we have developed

a simple multi-cluster wetting contact model, where clusters of differently spaced hexa-lattice of pillars are randomly distributed accordingly to Eq. (S6), and where one assumes that CB occurs for $L_{\mathrm{p}} < L_{\mathrm{th}}(p)$, and the W regime occurs elsewhere. Therefore, once determining for the hexa-lattice the normalized contact areas $A_{LV,()}$, $A_{LS,()}$ and $A_{SV,()}$, where LV, LS and SV is for liquid-air, liquid-solid and solid-air interface, and where () is to indicate the interface in the CB and W contact condition (relations dropped here for simplicity), the total wet contact area can be calculated with

$$\frac{A_{LS}(p_0)}{A_0} = \int_0^{L_{th}(p_0)} dL_{\mathrm{p}}\, P(L_{\mathrm{p}}) A_{LS,CB}(L_{\mathrm{p}}) + \int_{L_{th}(p_0)}^{\infty} dL_{\mathrm{p}}\, P(L_{\mathrm{p}}) A_{LS,W}(L_{\mathrm{p}})$$

and similarly for the liquid-air and solid-air interfaces. This provides, upon using Eqs. (S1-S3), the wetting predictions reported in Fig.SS3(A3 - top). In particular, for our nanotexture, a nominal CB regime is expected up to pressures much larger than those obtained in the experiments because of the sloshing action, i.e. a complete W transition occurs starting from order 10^5 Pa pressures.

In order to verify the prediction of the statistical model above, we have performed deterministic numerical resolution of the Young-Laplace differential equation which reads (neglecting high order terms in the local droplet mean curvature radius, which is the case here)

$$\Delta u(\mathbf{x}) - \frac{\mathrm{p}_0}{2\gamma_{LV}} = 0, \qquad (S7)$$

where p_0 is the droplet squeezing pressure and γ_{LV} the water surface tension. In the model, the pillars are assumed with same height (consistently with the real nano-pillar forest surface), but randomly distributed on the substrate. Several realizations of the virtual prototypes (both numerically generated or experimentally measured, see e.g. Fig.1(A.2- A4) and Fig.SS3(A.2)) have been simulated. In particular, in Eq. (S7) $u(\mathbf{x})$ is the droplet separation field measured from the (common) pillar top height. Eq. (S7) is thus solved on a periodic forest system (with periodicity boundary conditions, see Fig.1(A.3)), considering $u = 0$ on the pillars (top) edges, adopting mesh refining close to the pillar edges. The results were considered in the CB state if over none of the simulation triple lines the contact angle between pillar and water surface was overcoming the thermodynamic value. The simulations, performed within Mathematica (see an example of results in Fig.SS3(A.2)), confirmed that no CB to W transition was occurring for pressures below 10^5 Pa order, in agreement with the simple multi-cluster statistical model provided above. At a squeezing pressure of 2 kPa (quite above the maximum sloshing pressure in the experiments, see below), the droplet penetration field for one pillar forest realization is reported in Fig.SS3(A.2) (penetration exaggerated for easy of visualization).

By using Eq. (S3), the effective contact angle provided by the nanotexture is $\approx 123°$, and can thus be considered constant in our experiments. Indeed, considering $\beta_{\mathrm{LS}}^{\mathrm{N}} = \beta_{\mathrm{LV}}^{\mathrm{N}} = 0$, $\alpha_{\mathrm{LV}}^{\mathrm{N}} \approx 0.43$ (measured) and $\alpha_{\mathrm{LS}}^{\mathrm{N}} + \alpha_{\mathrm{LV}}^{\mathrm{N}} \approx 1$, this leads to an effective contact angle on the lower magnification (say, the microscale)

$$\cos\theta^{\mathrm{M}} = \cos\theta_0 \alpha_{\mathrm{LS}}^{\mathrm{N}} - \alpha_{\mathrm{LV}}^{\mathrm{N}}$$

obtaining $\theta^{\mathrm{M}} \approx 123°$ and $\gamma_{\mathrm{LS}}^{\mathrm{M}} = -\gamma_{\mathrm{LV}}\cos\theta^{\mathrm{M}} + \gamma_{\mathrm{SV}}^{\mathrm{M}}$, with

$$\gamma_{\mathrm{SV}}^{\mathrm{M}} = r_{\mathrm{sub}}^{\mathrm{N}}\gamma_{\mathrm{SV}},$$

where ($r_{\mathrm{sub}}^{\mathrm{N}} \approx 22.8$).

Therefore, if one considers the surface only made by the nano-texture, the total surface energy of a droplet (larger than representative nano-texture size) deposited onto the substrate is

$$U = -\gamma_{\mathrm{LV}}\cos\theta^{\mathrm{M}} A_{\mathrm{SL}} + r_{\mathrm{sub}}^{\mathrm{N}}\gamma_{\mathrm{SV}} A_0 + \gamma_{\mathrm{LV}} A_{\mathrm{LV}}, \qquad (S8)$$

with θ^{M} independent of the droplet squeezing pressure for pressures lower than about10^5 Pa.

S3. Effective wetting properties for the micro-textured surface

In this section we report the novel computational wetting model for the investigation of a three-dimensional random surface. We stress that the model reported in the following can be applied to any roughness topography, both with random or ordered geometries, and as such is applied to the simulation of the microtextured surface adopted in this research.

As stressed in Section S1, to integrate out the micro-texture contribution to the wetting dynamics when observing the system at lower magnifications, we use the effective equations (S1-S3) over the micro-texture. Here we calculate $\alpha_{\mathrm{LS}}^{\mathrm{M}}$ and $\alpha_{\mathrm{LV}}^{\mathrm{M}}$, to be used in Eqs. (S1-S3), by studying the wetting contact dynamics occurring on the microtexture. The total (micro-) system energy is

$$\frac{U}{\gamma_{\mathrm{LV}}} = A_{\mathrm{l}} - \cos\theta^{\mathrm{eff}} A_{\mathrm{c}} + \frac{p}{\gamma_{\mathrm{LV}}} u A_0 + \frac{\gamma_{\mathrm{SV}}^{\mathrm{eff}}}{\gamma_{\mathrm{LV}}} A_{\mathrm{tot}} \qquad (S9)$$

where A_l is the liquid free surface, A_c is the wet solid surface, A_{tot} is the total solid-phase surface. $\cos\theta^{\mathrm{eff}}$ and $\gamma_{\mathrm{SV}}^{\mathrm{eff}}$ are the contact angle and solid-vapor surface tension, respectively, as oberved on the microtextured surface. Furthermore,

$$dA_{\mathrm{l}} = \left(1 - f_{\mathrm{c}}(\mathbf{x})\right)\sqrt{1 + |\nabla u|^2}$$

$$dA_{\mathrm{c}} = f_{\mathrm{c}}(\mathbf{x})\sqrt{1 + |\nabla u|^2}$$

where $u(\mathbf{x})$ is the droplet separation measured from the average roughness plane (i.e. $\langle d(\mathbf{x})\rangle = 0$), and where $f_{\mathrm{c}}(\mathbf{x})$ is the wetting contact index defined as

$$f_{\mathrm{c}}(\mathbf{x}) = e^{-\frac{u(\mathbf{x}) - d(\mathbf{x})}{\rho}},$$

with $d(\mathbf{x})$ the surface roughness and ρ a characteristic length describing the distance over which the fluid-solid contact occurs.

In the case the nanotexture *is* included on the top of the microtexture (see e.g. Fig.1(B.2 - left)), $\cos\theta^{\mathrm{eff}} = \cos\theta^{\mathrm{M}}$ and $\gamma_{\mathrm{SV}}^{\mathrm{eff}} = r_{\mathrm{sub}}^{\mathrm{N}}\gamma_{\mathrm{SV}}$ are provided in Section S2, whereas in the case the nanotexture *is not* included $\cos\theta^{\mathrm{eff}} = \cos\theta_0$ and $\gamma_{\mathrm{SV}}^{\mathrm{eff}} = \gamma_{\mathrm{SV}}$ as given by the pristine PP substrate. Either cases (pristine PP and nanotextured PP) do not show pressure dependence in the terms $\cos\theta^{\mathrm{eff}}$and $\gamma_{\mathrm{SV}}^{\mathrm{eff}}$ wetting dynamics, therefore Eq. (S9) can be simplified to

$$\frac{U}{\gamma_{\mathrm{LV}}} = A_{\mathrm{l}} - \cos\theta^{\mathrm{eff}} A_{\mathrm{c}} + \frac{p}{\gamma_{\mathrm{LV}}} u A_0. \qquad (S10)$$

Eq. (S10) is numerically minimized with the finite element method in the variable $u(\mathbf{x})$ within the FEniCS software environment, where $\mathbf{x} = (x, y)$ is the plane of the nominally flat substrate, under the constraint $u(\mathbf{x}) \geq 0$ (no water intrusion in the solid phase), at constant applied droplet pressure *p*. The computational domain, a square periodic in the -*x* and -*y* directions with periodicity lengths $L_{0x} = L_{0y} = L_0$ (see Fig.1(B.4) and Fig.SS3B), is discretized with an unstructured mesh of Lagrangian triangular elements of order one (see Fig.SS3(B.2)). The mesh is recursively hierarchically refined close to the triple lines and, correspondingly, the total interface free energy minimized at decreasing values of the characteristic length ρ. The solver adopted for the non-linear constrained minimization is the TRON algorithm, active-set Newton trust region method for bound-constrained minimization. TRON is made available by the Tao (Toolkit for Advance Optimization) library within the PETSc (Portable, Extensible Toolkit for Scientific Computation) solver in FEniCS (https://fenicsproject.org/).

Once the equilibrium configuration is computed at a given droplet pressure *p*, we determine $\alpha_{\mathrm{LV}}^{\mathrm{M}} = A_{\mathrm{l}}/A_0$ and $\alpha_{\mathrm{LS}}^{\mathrm{M}} = A_{\mathrm{c}}/A_0$ to be used in the effective equations (S1-S3) over the micro-textured surface. The predicted effective wetting parameters, with and without nanoscale texture, are reported in Fig.2(A.2).

In particular, (only) for the case of micro-textured surface a breakdown of the simulations, for the different surface realizations, occurs at squeezing pressures ≈180 Pa (in between ≈150 and ≈200 Pa for the set of run simulations); this suggests that the numerical algorithm is not able to handle the abrupt variation of equilibrium solution across such pressure values, as a consequence of, possibly, a Wenzel transition. We will show in Supplementary Section S4 (see also Fig.SS5 where we show the comparison between theory and experiments) that, very interestingly, a Wenzel transition is exactly the phenomenon occurring when the squeezing pressure achieves such limiting value on micro-textured surface. Furthermore, in order to model the wetting for pressure above the Wenzel transition pressure, we just consider no free-droplet surface in Eq. (S3), leading to a predicted effective contact angle in the Wenzel regime to be ≈95°. The latter result is in very good agreement with experiments, see Fig.SS5. In the CB regime, instead, the calculated effective contact angle for the micro-textured surface is ≈141°, with relatively small dependence with the squeezing pressure (until the W transition), see Fig.SS3B.

Finally, the case where the nano-texture is superposed to the micro-texture (hierarchical surface) shows a stable CB regime in the range of tested squeezed pressures, see Fig.2(A.2). Thus, very interestingly, the addition of nanotexture strongly affects the wetting dynamics occurring on the substrate, leading to a stable effective contact angle of ≈155°, see the simulation results of Fig.2(A.2). Again, this is in very good agreement with the experimental results, see Fig.SS5.

S4. Theory of droplet statics among two plates

In this section we report the theory developed for the static contact of a droplet among two nominally flat (chemically and physically) dissimilar surfaces. In particular, a droplet with volume V_0 and density $\Delta\rho + \rho_A$, where ρ_A is the surrounding out-of-droplet fluid density, is squeezed in between two rigid parallel nominally flat surfaces at distance h_0, as schematically reported in Fig.SS6.

The energy F of the system is given by the sum of the potential energy U_{g} and the surface energy U_{s}, with

$$U_{\mathrm{g}} = \pi\Delta\rho g \int dz\, zr^2(z)$$

and

$$U_{\mathrm{s}} = \gamma_{\mathrm{LA}}A_{\mathrm{L}} + \gamma_{\mathrm{LS_1}}^{\mathrm{eff}}A_{\mathrm{S_1}} + \gamma_{\mathrm{LS_2}}^{\mathrm{eff}}A_{\mathrm{S_2}} + \gamma_{\mathrm{S_1A}}^{\mathrm{eff}}A_{\mathrm{NS_1}} + \gamma_{\mathrm{S_2A}}^{\mathrm{eff}}A_{\mathrm{NS_2}}. \qquad (S11)$$

The mesoscopic mechanical equilibrium requires that

$$\gamma_{\mathrm{LA}} = \frac{\gamma_{\mathrm{SA}}^{\mathrm{eff}} + \gamma_{\mathrm{LS}}^{\mathrm{eff}}}{\cos\theta^{\mathrm{eff}}}, \qquad (S12)$$

where θ^{eff} is the effective droplet contact angle measured on the generic nominally smooth substrate. Thus, we write Eq. (S11) with (S12)

$$U_{\mathrm{s}} = \gamma_{\mathrm{LA}}\left[A_{\mathrm{L}} - \cos\theta_1^{\mathrm{eff}}A_{\mathrm{S_1}} - \cos\theta_2^{\mathrm{eff}}A_{\mathrm{S_2}}\right] + U_{\mathrm{s0}}^{\mathrm{eff}},$$

with $U_{\mathrm{s0}}^{\mathrm{eff}} = \gamma_{\mathrm{S_1A}}^{\mathrm{eff}}A_{\,1} + \gamma_{\mathrm{S_2A}}^{\mathrm{eff}}A_{\,2}$, where $A_{\,1}$ (A_2) is the bottom (top) apparent substrate area. Furthermore, we have that

$$A_{\mathrm{S_1}} = \pi \int dz\, r^2(z)\delta(z)$$

$$A_{\mathrm{S_2}} = \pi \int dz\, r^2(z)\delta(z-h_0),$$

where h_0 is the distance between the plates (and $\delta(z)$ is Dirac delta), and where

$$A_{\mathrm{L}} = 2\pi \int dz\, r\sqrt{1+r_{,z}^2}.$$

We make all lengths **dimensionless** with $l_0 = V_0^{1/3}$, and energies with $\gamma_{\mathrm{LA}}l_0^2$, leading to

$$F = B_o \int dz\, \pi zr^2(z) + \int dz\, 2\pi r\sqrt{1+r_{,z}^2} - \left(\cos\theta_1^{\mathrm{eff}}A_{\mathrm{S_1}} + \cos\theta_2^{\mathrm{eff}}A_{\mathrm{S_2}}\right) + U_{\mathrm{s0}}^{\mathrm{eff}}$$

or

$$F = \int dz\left[B_o\pi zr^2(z) + 2\pi r\sqrt{1+r_{,z}^2} - \cos\theta_1^{\mathrm{eff}}\pi r^2(z)\delta(z) - \cos\theta_2^{\mathrm{eff}}\pi r^2(z)\delta(z-h_0)\right] + U_{\mathrm{s0}}^{\mathrm{eff}},$$

where in $r(z)$ the dependence with z has been omitted for simplicity, and where $B_{\mathrm{o}} = \Delta\rho g l_0^2/\gamma_{\mathrm{LA}}$ is the Bond number. In order to minimize F with constrained droplet volume, we introduce a (dimensionless) Lagrange multiplier λ

$$F_V = F - \lambda(V-1), \qquad (S13)$$

where V is the dimensionless droplet volume, V

$$V = \pi \int dzr^2. \qquad (S14)$$

In order to determine the equilibrium configurations related to the functional Eq. (S12), we derive the Euler-Lagrange equation from (S13) leading to the droplet shape differential equation

$$\sqrt{1+r_{,z}^2} - \frac{\partial}{\partial z}\left(r\frac{r_{,z}}{\sqrt{1+r_{,z}^2}}\right) + B_{\mathrm{o}}zr - \lambda r = 0 \qquad (S15)$$

and boundary conditions (BCs)

$$\frac{r_{,z}(h_0)}{\sqrt{1+r_{,z}^2(h_0)}} - \cos\theta_2^{\mathrm{eff}} = 0 \qquad (S16)$$

$$\frac{r_{,z}(0)}{\sqrt{1+r_{,z}^2(0)}} + \cos\theta_1^{\mathrm{eff}} = 0. \qquad (S17)$$

The droplet equilibrium configuration is given by solving Eqs. (S15) (with $V = 1$) and (S16-17) with parameters $r(z)$, λ, h_0, B_o, θ_1^{eff} and θ_2^{eff}. Thus, once provided B_o, θ_1^{eff} and θ_2^{eff}, $r(z)$ and λ can be determined as a function of the separation h_0.

On the other side, when the droplet is upper-plate-free, we consider the Eqs. (S15) ($V = 1$) and (S17) (with $r(h_0) = 0$). Note that $r(h_0) \to 0$ implies that $\left(\cos\theta_2^{\text{eff}}\right)^2 \to 1$ and similar for the other case. Again, once provided B_o, θ_1^{eff}, $r(z)$ and λ can be determined as a function of the droplet height h_0.

It is now interesting to determine the force(s) acting on the plate(s). In particular, we have that the droplet mechanical equilibrium requires that

$$p(0)r(0)^2 - p(h_0)r(h_0)^2 = \frac{\Delta\rho V g}{\pi} + 2\gamma_{\text{LV}}(r(0)\sin\theta_1 - r(h_0)\sin\theta_2),$$

whereas by integrating the fluid momentum equation along the z-axis

$$p(0) - p(h_0) = \Delta\rho g h_0,$$

leading to

$$p(0)[r(0)^2 - r(h_0)^2] = -r(h_0)^2\Delta\rho g h_0 + \frac{\Delta\rho V g}{\pi} + 2\gamma_{\text{LV}}(r(0)\sin\theta_1 - r(h_0)\sin\theta_2)$$

$$p(h_0)[r(0)^2 - r(h_0)^2] = -r(0)^2\Delta\rho g h_0 + \frac{\Delta\rho V g}{\pi} + 2\gamma_{\text{LV}}(r(0)\sin\theta_1 - r(h_0)\sin\theta_2).$$

Therefore, the total force acting on the bottom plate (assumed positive if repulsive) is

$$F_{\text{N}}(0)/\pi = p(0)r(0)^2 - \gamma_{\text{LV}}2r(0)\sin\theta_1$$

$$= \frac{r(0)^2}{r(0)^2 - r(h_0)^2}\left[-r(h_0)^2\Delta\rho g h_0 + \frac{\Delta\rho V g}{\pi} + 2\gamma_{\text{LV}}(r(0)\sin\theta_1 - r(h_0)\sin\theta_2)\right] - \gamma_{\text{LV}}2r(0)\sin\theta_1$$

and

$$F_{\text{N}}(h_0)/\pi = p(h_0)r(h_0)^2 - \gamma_{\text{LV}}2r(h_0)\sin\theta_2$$

$$= \frac{r(h_0)^2}{r(0)^2 - r(h_0)^2}\left[-r(0)^2\Delta\rho g h_0 + \frac{\Delta\rho V g}{\pi} + 2\gamma_{\text{LV}}(r(0)\sin\theta_1 - r(h_0)\sin\theta_2)\right] - \gamma_{\text{LV}}2r(h_0)\sin\theta_2.$$

In dimensionless units ($\gamma_{\text{LA}} l_0$) we have

$$F_{\text{N}}(0) = B_o \frac{r_0^2\left(1 - \pi r_{h_0}^2 h_0\right)}{r_0^2 - r_{h_0}^2} + 2\pi r_{h_0} r_0 \frac{r_{h_0}\sin\theta_1 - r_0\sin\theta_2}{r_0^2 - r_{h_0}^2} \qquad (S18)$$

and

$$F_{\text{N}}(h_0) = B_o \frac{r_{h_0}^2(1 - \pi r_0^2 h_0)}{r_0^2 - r_{h_0}^2} + 2\pi r_{h_0} r_0 \frac{r_{h_0}\sin\theta_1 - r_0\sin\theta_2}{r_0^2 - r_{h_0}^2}.$$

Clearly, $F_{\text{N}}(0) - F_{\text{N}}(h_0) = B_o$ which is the droplet-in-air buoyancy in dimensionless units. We note that when the top plate is missing, Eq. (S18) reduces to $F_{\text{N}}(0) = B_o$ as expected.

The upper plate is the hierarchical AAO modified by PFOTS **(**(1H,1H,2H,2H-Tridecafluorooctyl)trichlorosilane). The contact angle is approximately 150°, see Fig.SS4. In Fig.SS7 we report the force-displacement predictions for the case where the droplet is squeezed between the microtextured surface (bottom plate) and the superhydrophobic surface (top plate, see Fig.SS5). In particular, in Fig.SS7A we report the force (black line) and relative contact radius (blue line), predicted on the bottom plate, as a function of the top plate relative displacement. The contact radius *R* is made dimensionless with respect to the sessile contact radius R_0 the droplet would have if just deposited on the micro-textured surface.

The adopted relationship between the apparent contact angle on the micro-structured surface and the pressure, which models the CB to Wenzel transition on the micro-textured surface (see Supplementary Section S3) is reported in Fig.SS3B.

S5. Pull-off experiments with droplet

The adhesive force between water droplets and the surfaces of interest was measured by a high-sensitivity microelectromechanical balance system (Dataphysics DCAT11, Germany). 15 μL water droplets were attached to a hydrophobic metal ring, which was set to approach, contact, and leave the surfaces at a constant speed (Ref. 1). A CCD camera was used to monitor the approach and pull-off of the droplet; examples of the recorded images showing the evolution of the interaction

between a droplet and the surfaces under investigation is reported in Fig.SS8A. As shown in Fig.SS8B, once the water droplet is in contact with the surface, the force gradually increases and reaches the maximum before the droplet separates from the surface; the maximum values of the forces obtained for all surfaces were recorded in the curve as the adhesive forces. The residual mass of the water left on the surface is also recorded as reported in Fig.SS8C.

S6. Tribocurrent generation

Literature reports values of PP surface charging upon distilled water contact of $\rho_A \approx 6 * 10^{-6}$ $\mathrm{C/m^2}$. (Refs. 2, 3) Furthermore, in our triboelectric generator, the nominal tank flat area is $A_0 = 1.26 \cdot 10^{-3}\mathrm{m}^2$.
In the case of untextured surface, the true water/PP contact area A_c corresponds to A_0, leading to an ideal tribo-charge of order $Q_{unt} = A_0\rho_A = 0.76\,nC$. The latter is reported in Fig.2(D.1) as a reference value for ideal flat surface. Nevertheless, the drag-out dewetting (see Supplementary Section S9) is not completed at sloshing frequency of $3\mathrm{Hz}$ (i.e., the tank flat surface is not fully freed from a water film upon tank motion reversal), thus leading to a much-reduced tribocurrent generation, see Fig.2(D.1).
For the nanotextured surface the drag-out dewetting occurs, see Fig.2(D.2). In this case $A_c = A_0\alpha_c^N \approx 0.57A_0$, leading to a tribocurrent $Q_N \approx 0.43\mathrm{nC}$, in agreement with the experimental data, see Fig.2(D.1).
For the hierarchical texture, $A_c = A_0\alpha_c^H$, where $\alpha_c^H = \alpha_c^N * \alpha_c^m * a$, where $\alpha_c^m \approx 0.33$ and $a \approx 6.3$ (considering the largest microtexture wavelength to be sinusoidal, with same wavelength and amplitude, see e.g. Fig.1(B.1)). This leads to $Q_H \approx 0.89\mathrm{nC}$, in agreement with the experimental results.

S7. TENG experiments at different sloshing frequencies

Fig.SS9A shows a sequence of fluid motion images of the water-based triboelectrification power generation cycle for typical experimental parameters adopted during the testing: water-to-cylinder volume ratio 0.3, vibration frequency 3 Hz, and vibration amplitude 5 mm. The outputs generated in terms of current and voltage are shown in Figs.SS9B and SS9C respectively.

As already shown in our previous preliminary contributions (Ref. 4) describing the response of an optimized U-shaped triboelectrification device, the output current and voltage can show some degree of asymmetry. This might depend on the local conditions and the accuracy of the system design in terms of vibrations, as the system is particularly sensitive to the sloshing dynamics as discussed in the main manuscript. However, this aspect is not the focus of this contribution, whose main aim is to describe the remarkable ability of triboelectrification to finely detect the wetting dynamics.

S8. Weak non-linear theory of sloshing dynamics

In this section we report the model describing the sloshing dynamics characterizing the water-filled cylindrical-tank triboelectric generator. The contact pressure acting on the functionalized nominally-flat cylinder surfaces can be approximately predicted recurring to a weakly-nonlinear sloshing dynamics model of the partially filled tank. We note that the liquid free-surface (in partially filled tanks) can experience a wide cascade of energetic phenomena and related kinematics, the latter ranging from the simplest planar motion, to no-planar, rotational, quasi-periodic, random and lasting with free-surface fragmentation processes. A comprehensive review on the sloshing dynamics motions can be extracted by Ref. (5).

Here we focus on the determination of the average water squeezing pressure acting on the cylinder flat surfaces. To do so, we model the sloshing vibration dynamics within a Rayleigh approach by assuming the free-surface motion to be governed by a simple two-dimensional kinematics, where the free surface is approximated to a flat tilting surface, with rotation axis perpendicular to the cylinder tank axis of symmetry, see Fig.SS10(A.1). The application of the mass conservation (volume conservation in this case) leads the free-surface kinematics to be described by just one free Lagrangian parameter, which we consider to be the tilting angle φ of the free-surface with respect to the cylinder axis (with $\alpha = tan(\varphi)$).

In the following we report the main equations describing the surface kinematics. In particular, by assuming lengths (such as the cylinder tank axial length, L_0) **dimensionless** with respect to the cylinder radius, R_c, the relation between the center of mass $\mathbf{x}_G = (x_G, z_G)$ as a function of the tilting angle $\alpha = tan(\varphi)$ is calculated as follows:

$$z_{\mathrm{G}} = \frac{-\dfrac{2\sin^{-}1(1+\alpha l_1)+\pi}{8\alpha} + \dfrac{(2\alpha^3 l_1^3 + 6\alpha^2 l_1^2 + \alpha l_1 - 3)\sqrt{\alpha(-l_1)(\alpha l_1 + 2)}}{12\alpha}}{V}$$

if $2 + l_1\alpha < L_0\alpha$ and $l_1 < 0$

$$z_{\mathrm{G}} = \frac{-\dfrac{\pi}{4\alpha}}{V}$$

if $2 + l_1\alpha < L_0\alpha$ and$l_1 \geq 0$

$$z_{\mathrm{G}} = -\frac{\big(1+\alpha(l_1 - L_0)\big)(2(1+\alpha(l_1-L_0))^2 - 5)\sqrt{1-(1+\alpha(l_1-L_0))^2}}{12\alpha V} +$$
$$+\frac{\sin^{-1}(1+\alpha(l_1-L_0)) - \sin^{-1}(1+\alpha l_1)}{4\alpha V} + \frac{(\alpha l_1 + 1)(2\alpha^2 l_1^2 + 4\alpha l_1 - 3)\sqrt{\alpha(-l_1)(\alpha l_1 + 2)}}{12\alpha V}$$

if $2 + l_1\alpha \geq L_0\alpha$ and $l_1 < 0$

$$z_{\mathrm{G}} = -\frac{\big(1+\alpha(l_1 - L_0)\big)(2(1+\alpha(l_1-L_0))^2 - 5)\sqrt{1-(1+\alpha(l_1-L_0))^2}}{12\alpha V} + \frac{\sin^{-1}\big(1+\alpha(l_1-L_0)\big) - \dfrac{\pi}{2}}{4\alpha V}$$

if$2 + l_1\alpha \geq L_0\alpha$ and $l_1 \geq 0$,

and

$$x_{\mathrm{G}} = \frac{(\alpha l_1 + 1)\big(1+\alpha(l_1-L_0)\big)\tan^{-1}\left(\dfrac{\alpha(-l_1)-1}{\sqrt{1-(\alpha(-l_1)-1)^2}}\right) - \pi(2+\alpha l_1)\big(2+\alpha(l_1-L_0)\big)}{2\alpha^2 V} +$$
$$+\frac{\begin{array}{c}6\pi(\alpha l_1+1)\big(1+\alpha(l_1-L_0)\big) + 12\pi(\alpha(-L_0)+2\alpha l_1+3)\\ -3\sin^{-1}(\alpha l_1+1) - \dfrac{3\pi}{2} - b_1\sqrt{\alpha(-l_1)(\alpha l_1+2)}\end{array}}{24\alpha^2 V}$$

if$2 + l_1\alpha < L_0\alpha$ and $l_1 < 0$

$$x_{\mathrm{G}} = \frac{\left\{\dfrac{\pi(-4\alpha L_0 + 8\alpha l_1 + 11)}{8\alpha^2} - \dfrac{\pi(2+\alpha l_1)\big(2+\alpha(l_1-L_0)\big)}{2\alpha^2}\right\}}{V}$$

if $2 + l_1\alpha < L_0\alpha$ and $l_1 \geq 0$

$$x_{\mathrm{G}} = \frac{12(\alpha l_1+1)\big(1+\alpha(l_1-L_0)\big)\left(\tan^{-1}\left(\dfrac{\alpha(-l_1)-1}{\sqrt{1-(\alpha(-l_1)-1)^2}}\right) - \tan^{-1}\left(\dfrac{\big(1-\alpha(l_1-L_0)\big)}{\sqrt{1-(1-\alpha(l_1-L_0))^2}}\right)\right)}{24\alpha^2 V}$$
$$+\frac{a_1\sqrt{1-(1+\alpha(l_1-L_0))^2} - b_1\sqrt{\alpha(-l_1)(\alpha l_1+2)} + 3\sin^{-1}(\alpha(-L_0)+\alpha l_1+1) - 3\sin^{-1}(\alpha l_1+1)}{24\alpha^2 V}$$

if $2 + l_1\alpha \geq L_0\alpha$ and $l_1 < 0$

$$x_{\mathrm{G}} = \left\{\frac{12(\alpha l_1+1)(\alpha L_0 - \alpha l_1 - 1)\tan^{-1}\left(\dfrac{\big(1-\alpha(l_1-L_0)\big)}{\sqrt{1-(1-\alpha(l_1-L_0))^2}}\right) + a_1\sqrt{1-(1+\alpha(l_1-L_0))^2}}{24\alpha^2 V}\right.$$
$$\left.+\frac{-6\pi(\alpha l_1+1)\big(1+\alpha(l_1-L_0)\big) + 3\sin^{-1}\big(1+\alpha(l_1-L_0)\big) - \dfrac{3\pi}{2}}{24\alpha^2 V}\right\}$$

if $2 + l_1\alpha \geq L_0\alpha$ and $l_1 \geq 0$,

where V is considered fixed when assuming the liquid to be incompressible, with the volume fraction of cylinder filled with water $V_{\mathrm{frac}} = V/(\pi L_0)$. Furthermore, we have that

$$a_1 = \alpha(2\alpha^2(L_0 - l_1)^2(L_0 + l_1) - 2\alpha(L_0 - l_1)(L_0 + 3l_1) - 7L_0 + 19l_1) + 15$$
$$b_1 = (-4\alpha L_0(\alpha^2 l_1^2 + 2\alpha l_1 + 3) + 2\alpha^3 l_1^3 + 6\alpha^2 l_1^2 + 19\alpha l_1 + 15).$$

and l_1 is given by:

$$V = \frac{\pi}{\alpha} + \frac{(1+\alpha l_1)\cos^{-1}(1+\alpha l_1)}{\alpha} - \frac{\big(3+\alpha l_1(2+\alpha l_1)\big)\sqrt{\alpha(-l_1)(2+\alpha l_1)}}{3\alpha} + \pi\left(L_0 - \left(\frac{2}{\alpha}+l_1\right)\right)$$

if $2 + l_1\alpha < L_0\alpha$ and $l_1 < 0$,

$$V = \frac{\pi}{\alpha} + \pi\left(L_0 - \left(\frac{2}{\alpha}+l_1\right)\right)$$

if $2 + l_1\alpha < L_0\alpha$ and $l_1 \geq 0$,

$$V = \frac{[2+(1+\alpha(l_1-L_0))^2]\sqrt{1-(1+\alpha(l_1-L_0))^2}}{3\alpha} - \frac{(2+(1+\alpha l_1)^2)\sqrt{\alpha(-l_1)(2+\alpha l_1)}}{3\alpha} + \frac{(1+\alpha l_1)\cos^{-1}(1+\alpha l_1) + \big(1-\alpha(l_1-L_0)\big)\cos^{-1}\big(1+\alpha(l_1-L_0)\big)}{3\alpha}$$

if $2 + l_1\alpha \geq L_0\alpha$ and $l_1 < 0$,

$$V = \frac{\Big(3-\alpha(L_0-l_1)\big(2+\alpha(l_1-L_0)\big)\Big)\sqrt{\alpha(L_0-l_1)\big(2+\alpha(l_1-L_0)\big)}}{3\alpha} - \frac{\big(1+\alpha(l_1-L_0)\big)\cos^{-1}\big(1+\alpha(l_1-L_0)\big)}{\alpha}$$

if $2 + l_1\alpha \geq L_0\alpha$ and $l_1 \geq 0$.

We observe that, by varying the tilting angle φ, the fluid center of mass $\mathbf{x}_G$ decribes a trajectory $\mathbf{x}_G(\alpha)$, whose curvilinear abscissa we name $s(\alpha)$. In the model presented here, mass conservation implies that the trajectory tangent $d\mathbf{x}_G/ds$ is given with $dz_G(s)/dx_G(s) = \tan\alpha$ (it can be easily proved by evaluating the variation of $(\mathbf{x}_G V)_{|s}$). Finally, assuming all the liquid inertia concentrated in the center of mass, the linear momentum conservation imposes that

$$\ddot{s}(t) + c\dot{s}(t) + g\sin\alpha(t) + \ddot{x}_0(t)\cos\alpha(t) = 0, \quad \text{(S19)}$$

where $\alpha(t) = \alpha\big(s(t)\big)$, and where again lenghts are made dimensionless using the cylinder radius as reference length, and time using $(2\pi f)^{-1}$, where f is the excitation frequency of the external shaking motion of the tank which reads (in dimensionless units) $x_0(t) = x_0\sin(t)$. g is the dimensionless gravitational acceleration $[gR^{-1}(2\pi f)^{-2}]$, assumed perpendicular to the cylinder axis. c is a (dimensionless) fluid damping parameter which can be evaluated as $c \approx \nu/(2\pi f h_0^2)$, where ν is the fluid kinematic viscosity, and where h_0 is representative of the sheared fluid thickness. In our system, $2\pi f c \approx 10^{-2}$ to $10^{-1}\mathrm{s}^{-1}$ for a tank filling ratio of 30% (value adopted in the experiments).

Eq. (S19) is evaluated numerically, upon which the (dimensionless) fluid squeezing load acting on the tank flat surfaces can be calculated, within the approximated model above, as follows

$$F_w(t) = \left[g\cos\alpha(t) + \frac{\dot{s}(t)^2}{r\big(s(t)\big)}\right]\sin\alpha(t),$$

where $r(s)$ is the curvature radius of the trajectory at abscissa s

$$r(s)^{-1} = \frac{d\tan\alpha(s)}{ds}\big(\cos\alpha(s)\big)^2.$$

Finally, the fluid squeezing pressure, with dimensions, for our system is $p_w = \frac{1}{2}c_w\rho\bar{v}^2$, where $\bar{v} = 2fL_0$ is the average sloshing speed and $c_w = 2\pi^2 F_w R L_0^{-1} V_{\text{frac}}$ is the dimensionless wall impact coefficient (V_{frac} is the tank filling ratio). Results for the case of $f = 3\ Hz$ are reported in Fig.2(B.2), whereas the sloshing trajectories at varying filling ratios are reported in Fig.SS10(A.2).

S9. Film dewetting by drag-out mechanics

The sloshing motion causes the bulk waterfront to be squeezed/retrieved onto/from the tank flat surfaces, nominally at the same frequency of the shaking motion. However, after this bulk detachment of the waterfront, a thin water film might be left on the flat surfaces. Indeed, the dewetting time, necessary for this residual fluid film to be drag-out from the flat surfaces, is not necessarily matching the sloshing frequency, and, as such, it needs to be evaluated by separately studying the drag-out dewetting dynamics of the water front on the flat cylinder surfaces. Interestingly, if the characteristics time of the drag-out dynamics is larger than the characteristics

sloshing time, then the water film is never dewetting from the PP surfaces, leading to no triboelectric charging in the system.

Here we evaluate the drop off thickness of the water on the PP plate assuming the confined liquid film removal to be described within the so-called drag-out problem, see Fig.SS11, where an inclined plate is withdrawn from a pool of liquid and one needs to calculate the thickness of the film clinging to the plate. For a vertical plate, this setting was examined by Landau and Levich (1942), a theory later extended by Wilson (1982) to the case of a plate inclined at an arbitrary angle (so called LLW solution). Here we use the non-LLW solutions to describe the general case of non-perfectly wetting liquid, where the film thickness is predicted to be $h_{\min} = 3^{1/2}\sqrt{v_0\eta/(\rho g)}$, with $v_0 = 2Rf$ the drag-out speed. Thus, for our system, we get $h_{\min} = 0.110$, 0.156, and $0.192\mathrm{mm}$ at the different sloshing frequencies of the experiments, respectively, 1, 2 and 3 Hz.

Once knowing the film thickness $h_{\min}$ during drag-out, we now need to determine whether this water layer is energetically stable or either a dewetting transition is more favorable to occur, as well its characteristics dewetting time. To do so, we make use of the thin film (lubrication) theory. In particular, we calculate first the disjoining pressure $\Pi(h)$ acting in the film (which can be obtained from the Gibbs free energy $\mathrm{G}(h)$ of the film, with $\Pi(h) = -d\mathrm{G}(h)/dh$) whose polar and apolar contributions are

$$\Pi(h) = 2S_{\mathrm{AP}}\frac{d_0^2}{h^3} + \frac{S_{\mathrm{P}}}{l}e^{\frac{d_0-h}{l}}, \qquad (\mathrm{S20})$$

where $d_0 = 0.158\ \mathrm{nm}$ is the Born repulsion length and l the correlation length for a polar fluid, which for water is about $l_{\mathrm{W}} = 0.6\ \mathrm{nm}$. S_{P} and S_{AP} are the polar and apolar components of the spreading coefficient $S = S_{\mathrm{P}} + S_{\mathrm{AP}}$. We also have that $S = \gamma_{\mathrm{SV}} - \gamma_{\mathrm{SL}} - \gamma_{\mathrm{LV}} = \gamma_{\mathrm{LV}}(\cos\theta - 1)$, where the thermodynamic contact angle of water on pristine PP $\theta = 102°$. The apolar component S_{AP} can be derived from the effective Hamaker constant of the interface air-water-solid (PP is the solid), A_{GWS}, with $S_{\mathrm{AP}} = -A_{\mathrm{GWS}}/(12\pi d_0^2)$. For contact of two dissimilar materials in the presence of a third media $A_{\mathrm{GWS}} = A_{\mathrm{GS}} + A_{\mathrm{WW}} - A_{\mathrm{GW}} - A_{\mathrm{WS}} \approx A_{\mathrm{WW}} - A_{\mathrm{WS}}$. Since $A_{\mathrm{WS}} \approx \sqrt{A_{\mathrm{WW}}A_{\mathrm{SS}}}$ one has

$$A_{\mathrm{GWS}} \approx \sqrt{A_{\mathrm{WW}}}\left(\sqrt{A_{\mathrm{WW}}} - \sqrt{A_{\mathrm{SS}}}\right).$$

We note further that $A_{\mathrm{SS}} = 24\pi d_0^2\gamma_{\mathrm{S}}$. For polypropilene, $\gamma_{\mathrm{S}} = 0.031\mathrm{N/m}$, whereas for water $A_{\mathrm{WW}} = 4.38 \times 10^{-20}\mathrm{Nm}$, leading to $A_{\mathrm{SS}} = 5.83 \times 10^{-20}\mathrm{Nm}$ and to $A_{\mathrm{GWS}} = -6.75 \times 10^{-21}\mathrm{Nm}$. Thus, we get $S_{\mathrm{AP}} = 0.0072\mathrm{N/m}$ and $S_{\mathrm{P}} = -0.094\mathrm{N/m}$ (Ref. 6). Therefore, in the disjoining pressure the apolar component is stabilizing ($S_{\mathrm{AP}} > 0$) whereas the polar is destabilizing ($S_{\mathrm{P}} < 0$) the water film thickness. In Eq. (S20), for h larger than $\approx 0.265\ \mathrm{nm}$ we have $d\Pi(h)/dh > 0$, i.e. the water films are nominally unstable in our case.

If we assume the film breakdown occurs in correspondence of the discontinuities of the tank flat surfaces, we can now easily estimate the time needed for the film to retrieve from the flat surfaces. Within lubrication hydrodynamics this characteristic time t_0 reads

$$t_0 \approx \frac{6s_0^2\eta}{h_{\min}^2\left(p_0 - 2h_{\min}^{-1}\gamma_{\mathrm{LV}}\cos\theta_{eff}\right)},$$

with $p_0 = gs_0\rho$ and $s_0 = 2R$, and where θ_{eff} is the apparent water contact angle on the surface.

It is interesting to compare the case of dewetting occurring on the pristine PP surface (untextured surface) with the nanotextured surface. Indeed, since the water-PP contact area is reduced in the nanotextured case with respect to the untextured case, one would expect the surface charging to be higher for the untextured surface. Nevertheless, we get that for the case of $\theta_{eff} = 102°$ (pristine PP surface) the water film dewets the PP surface only for frequencies lower than $\approx 0.5\mathrm{Hz}$, whereas for $\theta_{eff} = 123°$ (nanotextured surface) for frequencies lower than $\approx 3.4\mathrm{Hz}$, see Fig.2(D.2). Thus, for the untextured case, the water is not able to fully dewet the PP surface in the range of sloshing frequencies under investigation, remarkably leading to a smaller charge accumulation with respect to the nanotextured case.

S10. TENG experiments at different surface energies

In a liquid, the variation of the surface tension can also drastically influence the Cassie-Baxter-to-Wenzel transition, thus the triboelectrification. This can be proved by simply adding surfactants

to change the surface tensions of water. Therefore, after analyzing the effect of different surfaces and sloshing frequencies as reported in Fig.2 and Fig.SS12, the effect of surfactant contamination was investigated. Fig.SS13 shows that the output current of water-based triboelectrification device is greatly related to the surfactant concentration. It could be observed that the current stays at the maximum level (~40 nA) when there is no surfactant concentration, and decreases to a lower level (~0.4 nA) once the surfactant is added to the system. Fig.SS13C presents the sensitivity of a water-based hierarchical triboelectrification device and a water-based smooth triboelectrification device in the detection of surfactants. It is shown that the output current of water-based hierarchical triboelectrification device decreases more drastically as the surfactant concentration is increased. Compared to a water-based smooth triboelectrification device, water-based hierarchical triboelectrification devices have a better sensitivity to detect the presence of surfactants as their addition can change the wetting state and the concentration of ions significantly. The decrease of the surface triboelectric charges of the water-based smooth triboelectrification device only contributes to an increase of the concentration of ions. In contrast to this, for the water-based hierarchical triboelectrification device the wetting state transition of the polymeric surface also affects triboelectric generation. So, the water-based hierarchical triboelectrification device shows superiority in the detection of surfactant.

With the surfactant concentration changing from 0 to 1μM, the generated current of the water-based hierarchical surface does not register particular reductions. When the surfactant concentration changes from 10 μM to 100 μM, the generated current marginally decreases. However, the generated current shows a faster decay when the surfactant concentration increases from 500 μM to 1 mM, see Fig.SS13C. Thus, the relationship between the output of water-based triboelectrification device and surfactant concentration could be divided into three wetting states (CB state, partially Wenzel state, and full Wenzel state) based on the magnitude of the current reduction as shown in Fig.SS13. In the low surfactant concentration (CB state), the polymer surface maintains superhydrophobicity during the wetting/dewetting cycles. The output current of the water-based hierarchical triboelectrification device has little change as time increase. The decrease of output current with the increase of surfactant concentration merely contributes to the increase of ions concentration. In the partially wetted state, part of the PP surface has been wetted, and the wetting state starts to transform the response from the Cassie-Baxter state to the Wenzel state. Since a permanent water film is formed, the output current of the water-based hierarchical triboelectrification device marginally decreases, then remains unchanged. In the full Wenzel state, the polymer surface is irreversibly wetted. The output current shows a faster decay during the contact-separation (wetting-dewetting) cycles. The Cassie-Baxter-to-Wenzel transition and the increase of ions concentration act synergistically to cause the change of current output.

S11. Theory of multiscale wetting film formation on rough surface

In this section we report a novel theory, based on the random walk process, for the prediction of the statistics of wetting film thickness on a generic random multiscale surface roughness. We observe that, upon deposition of a thin liquid film (with initial uniform thickness *h*) on a smooth rigid substrate, spinoidal dewetting occurs for $d\Pi(h)/dh > 0$, where $\Pi(h)$ is the disjoining pressure (see e.g. Supplementary Section S9). For our system (polypropilene), instability occurs for thin films with thickness larger than $h_{\mathrm{lim}} = 0.26\ \mathrm{nm}$ on an ideally smooth substrate.

We now consider the case of a thin uniform film of initial thickness v_0 on a rough rigid surface, characterized by a single wavelength q and amplitude $h = h(q)$, *i.e.* with surface roughness $h\sin(qx)$. In such a case, dewetting occurs accordingly to a thin film evolution equation for the amplitude (linearized model, see e.g. Ref. 7) which reads

$$v(t) = v_0 - hq^2(q^2 - k_c^2)^{-1}\left(1 - e^{-\frac{t}{\tau}}\right), \qquad (S21)$$

where $v(t)$ is the film thickness at time t, and τ is a time scale for dewetting ($\tau \propto (q^2 - k_c^2)^{-1}$). $k_c^2 = \Pi'(v_0)/\gamma_l$ is the critical spinoidal wavelength, obtained by linearizing the model around the initial film thickness value v_0. In the simplest picture, roughness-driven dewetting and spinoidal dewetting are the two competing mechanisms delivering the breakdown of an energetically unstable film ($d\Pi(h)/dh > 0$). We note however that the fastest dewetting mode is also strongly affected by the initial inhomogeneity of the film thickness [7]. To simplify the model, in the following we assume the

dewetting to occur independently at each roughness wavelength, consistently with the Gaussian nature of the surface roughness at lengthscales larger than the mean microcube size, see Fig.SS14.

We now consider the surface to be characterized by a random roughness $h(\mathbf{q})$, where $h(\mathbf{q}) = (2\pi)^{-2} \int d^2x\, h(\mathbf{x}) e^{-i\mathbf{q}\cdot\mathbf{x}}$ and $h(\mathbf{x})$ is the random surface roughness. We observe the surface at magnification $\zeta = q/q_0$ ($q = |\mathbf{q}|$), i.e. by ideally including roughness up to the frequency q (where $q_0 = 2\pi/L_0$ is a reference frequency, say the roughness lower cutoff frequency of the power spectral density, PSD, see Fig.3(A.1)). At this magnification ζ a fraction of the total surface A_0 surface (say $A_\mathrm{w}(\zeta)$) appears covered by a random distribution of film thicknesses with average volume $V_0 = v_0 A_0$, as a consequence of the dewetting due to the higher roughness wavelengths $\lambda > 2\pi/q$. $A_\mathrm{w}(\zeta)$ is the apparent wet surface area at magnification ζ, *i.e.* the wet area obtained when adding roughness up to the frequency $q = \zeta q_0$. At magnification ζ, the probability distribution of wetting film thickness is $p(q = \zeta q_0, v)$, with $A_\mathrm{w}(\zeta) = \int_0^\infty dv\, p(\zeta, v)$ and with $v(\mathbf{x}, q)$ the film thickness distribution. We now add a small amount of (random) surface roughness in the frequency range $[q, q + \Delta q]$, where $\Delta q = q_0 \Delta\zeta$ is the surface roughness frequency variation. Therefore, when we add this small amount of smaller scale roughness with wavenumber between q to $q + \Delta q$, $v(\mathbf{x}, q)$ randomly grows by an amount $\Delta v(\mathbf{x}, q)$. We assume $\Delta v(\mathbf{x}, q)$ to be the sum of an infinite number of wavevectors $\mathbf{q}$ in the range q to $q + \Delta q$ with homogeneously distributed phases, thus statistically independent wave-components. Therefore, $\Delta v(\mathbf{x}, q)$ is a Gaussian variable, with probability density function $\mathrm{p}_{\Delta v(\mathbf{x},q)}$ given by

$$\mathrm{p}_{\Delta v(\mathbf{x},q)}(\Delta v) = \frac{e^{-\frac{\Delta v^2}{2\langle \Delta v(\mathbf{x},q)^2\rangle}}}{\sqrt{\pi}\sqrt{2\langle \Delta v(\mathbf{x},q)^2\rangle}}. \tag{S22}$$

In order to calculate the probability density function $p(q + \Delta q, v)$ of the film thickness field $v(\mathbf{x}, q + \Delta q)$ when adding roughness in the range q to $q + \Delta q$, we can use Bayesian inference, resulting in

$$p(q + \Delta q, v) = \int_0^\infty dv'\, \mathrm{p}_{\Delta v(\mathbf{x},q)}(v - v') p(q, v'). \tag{S23}$$

Hereinafter we omit the dependence on the spatial variable $\boldsymbol{x}$. After some manipulation, we write (S23) as

$$\mathrm{p}(q + \Delta q, v) = \int_{-\frac{v}{(2\langle \Delta v(\mathbf{x},q)^2\rangle)^{1/2}}}^{\infty} dz\, \frac{e^{-z^2}}{\sqrt{\pi}} \mathrm{p}\left(q, v + z\sqrt{2\langle \Delta v(\mathbf{x}, q)^2\rangle}\right). \tag{S24}$$

We then expand $\mathrm{p}\left(q, v + z\sqrt{2\langle \Delta v(\mathbf{x}, q)^2\rangle}\right)$ in $\sqrt{2\langle \Delta v(\mathbf{x}, q)^2\rangle}$ to get

$$\mathrm{p}\left(q, v + z\sqrt{2\langle \Delta v(\mathbf{x}, q)^2\rangle}\right) = \mathrm{p}(q, v) + \frac{\partial \mathrm{p}(q, v)}{\partial v} \langle 2\Delta v(q)^2\rangle^{1/2} z + \frac{\partial^2 \mathrm{p}(q, v)}{\partial v^2} \frac{\langle 2\Delta v(q)^2\rangle z^2}{2} + o\left(\langle 2\Delta v(q)^2\rangle^{3/2} z^3\right),$$

and $\mathrm{p}(q + \Delta q, v)$ in Δq to get

$$\mathrm{p}(q + \Delta q, v) = \mathrm{p}(q, v) + \frac{\partial \mathrm{p}(q, v)}{\partial q} \Delta q + o(\Delta q^2).$$

In the limit $\Delta q \to 0$ (S24) becomes

$$\mathrm{p}(q, v) + \frac{\partial \mathrm{p}(q, v)}{\partial q} \Delta q = \int_{-\infty}^{\infty} dz\, \frac{e^{-z^2}}{\sqrt{\pi}} \left[\mathrm{p}(q, v) + \frac{\partial \mathrm{p}(q, v)}{\partial v} \langle 2\Delta v(q)^2\rangle^{1/2} z + \frac{\partial^2 \mathrm{p}(q, v)}{\partial v^2} \frac{\langle 2\Delta v(q)^2\rangle z^2}{2}\right]$$

which, after integration anddefining $\langle \Delta v(q)^2\rangle = \Delta q\, 2f(q)$, becomes

$$\frac{\partial p(q,v)}{\partial q} = f(q) \frac{\partial^2 p(q,v)}{\partial v^2}, \tag{S25}$$

where $v(q)$ is the diffusion term. (S25) is solved with the initial condition $p(q_0, v) = \delta(v - v_0)$, where v_0 is the initial average film thickness, and with $p(q, v = 0) = p(q, v \to \infty) = 0$. This leads to the solution

$$P(F, v) = \frac{e^{-\frac{(v - v_0)^2}{4F}}}{2\sqrt{\pi F}} - \frac{e^{-\frac{(v + v_0)^2}{4F}}}{2\sqrt{\pi F}}, \tag{$S26$}$$

where $dF(q)/dq = f(q)$. The normalized projected wet area

$$\frac{A_\mathrm{w}}{A_0} = \int dv\, P(F, v) = \mathrm{erf}\left(\frac{v_0}{2\sqrt{F}}\right) \tag{$S27$}$$

and the volume of fluid conservation is satisfied

$$\int dv\, vP(F,v) = v_0.$$

We now need to calculate $\langle \Delta v(q)^2 \rangle$. From (S21)

$$v(q) = h(q)\frac{q^2}{q^2 - k_c^2}$$

leading to

$$\langle \Delta v(q)^2 \rangle = 2\pi q \left(\frac{q^2}{q^2 - k_c^2}\right)^2 C(q)\, dq = \Delta q\; 2f(q)$$

and to

$$F(q) = \int_0^q d\bar{q}\; \pi \bar{q} \left(\frac{\bar{q}^2}{\bar{q}^2 - k_c^2}\right)^2 C(\bar{q}).$$

If we consider the case where $\bar{q} \ll k_c$, then $F(q) = k_c^{-4} \int_0^q d\bar{q}\; \pi \bar{q}^5 C(\bar{q}) = k_c^{-4} m_4(q)/2$, *i.e.* proportional to the scale-dependent mean square curvature $m_4(q)$. The true normalized projected wet area is therefore

$$\frac{A_{\mathrm{w}}(q_1)}{A_0} = \int dv\; P(F(q_1), v) = \mathrm{erf}\left(\frac{v_0}{2\sqrt{F(q_1)}}\right) \qquad (S28)$$

with a probability distribution of film gap

$$P(q_1, v) = \frac{e^{-\frac{(v - v_0)^2}{4(q_1)}}}{2\sqrt{\pi(q_1)}} - \frac{e^{-\frac{(v + v_0)^2}{4(q_1)}}}{2\sqrt{\pi(q_1)}}, \qquad (S29)$$

where q_1 is the roughness high frequency cut-off.

In Fig.SS15 we report the epifluorescence optical acquisitions of the residual wet areas on the textured PP surfaces at different sloshing times (3Hz sloshing frequency). Rodhamine B is dissolved in water with concentration 0.01mg/ml. The acquisitions (lighting and image acquisition) have been triggered in a reduced set of time intervals, thus avoiding any significant photo-bleaching phenomenon to occur in the experiments. Comparisons between the measured residual intensity and the predicted results is terms of probability distribution of film gap are reported in the main text and Fig.3.

Fig.SS1.

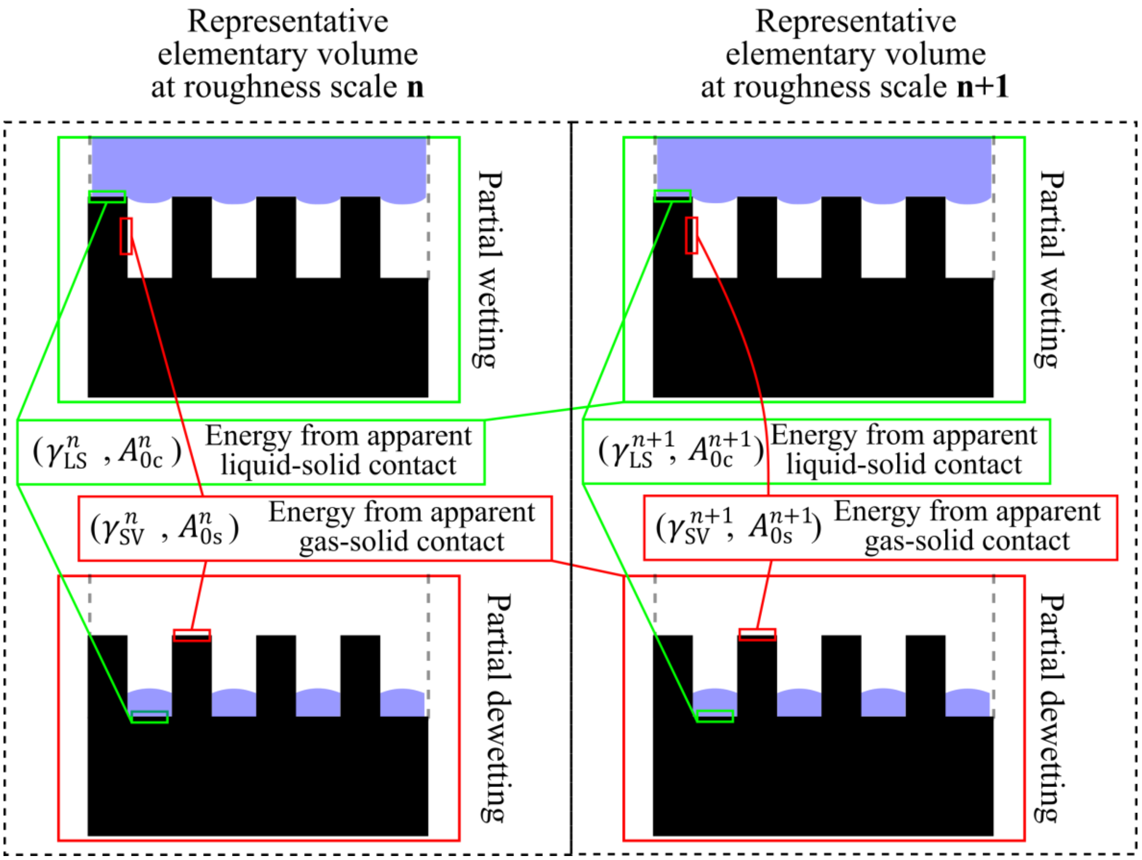

Schematic representation of the generic multiscale wetting process, involving different magnifications in the contact geometry (such as the length scale *n* and *n+1*, in the figure above). At each magnification, the contact can be in a partial wetted (green box) or de-wetted (red box) state.

Fig.SS2.

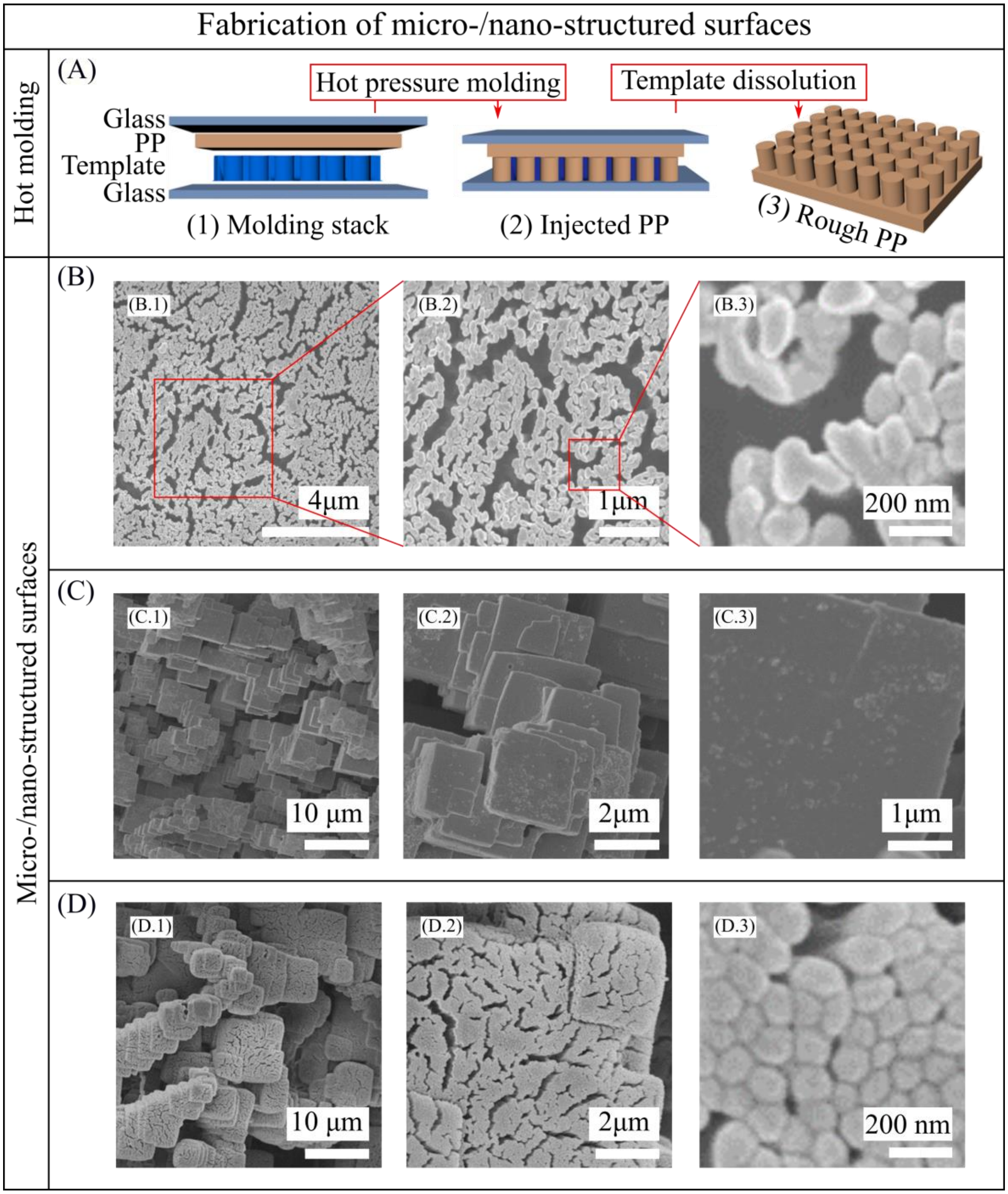

Schematics of the fabrication process of the different surface roughness on PP substrate. (A) a fabrication process of the PP based on a hot molding process. (B) FESEM images of the nanotextured PP, (C) microtextured PP and (D) the hierarchical PP. (D.3) FESEM micrographs of the nanopillar forest covering the micro-cubes.

Fig.SS3.

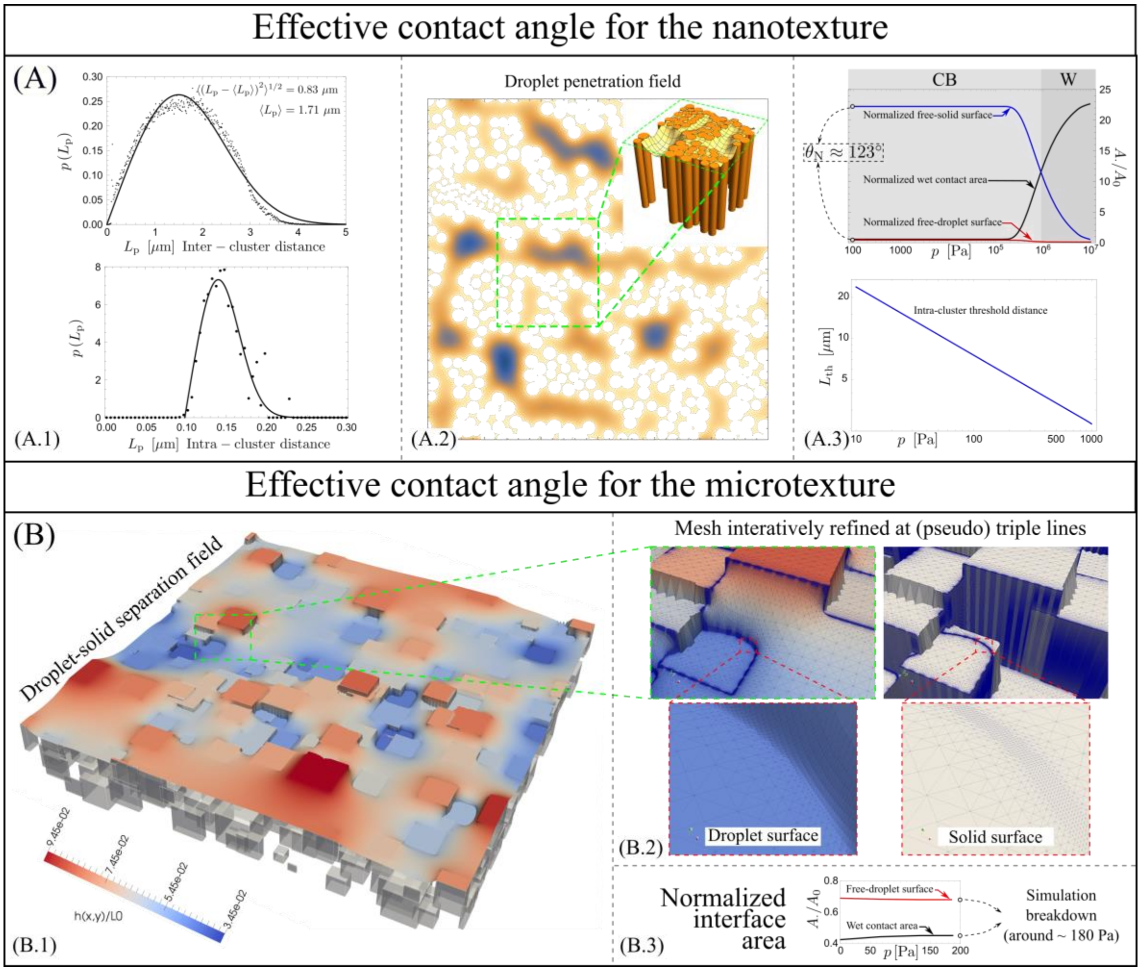

Wet contact simulation results. (A) The case of nanopillar surface, with indication of (A.1) inter- and intra-cluster distance probability density function. (A.2) Example of water penetration into the nanopillar forest with related three-dimensional representation (exaggerated penetration values). (A.3) effective contact angle (top) and intra-cluster threshold distance as a function of the water squeezing pressure. A Cassie-Baxter (CB) regime applies in the range of pressures of relevance for our application (see text), whereas the Wenzel regime is gradually experienced only for pressures larger than order 10^5 Pa, however the reversible wet contact area is constant, and given by the cumulative top pillars area. The inter-cluster threshold distance, as a function of water squeezing pressure, is the mean lattice spacing between clustered nanopillars at which the Wenzel state occurs. (B) The case of microtextured surface. (B.1) Example of water surface contacting the microtextured surface, with magnified view of the (B.2) water front and microtextured surface meshes. (B.3) Free-water surface and wet contact normalized surface area as a function of the squeezing pressure. The model breaks down upon reaching a certain amount of squeezing pressure.

Fig.SS4.

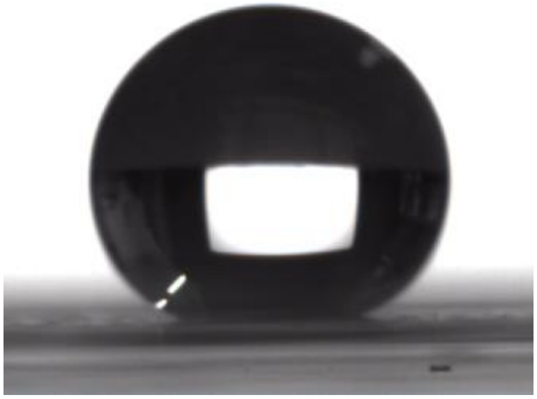

Contact angle of the upper plate (hydrophobic) in the droplet squeeze experiment. The contact angle is approximately 150°.

Fig.SS5.

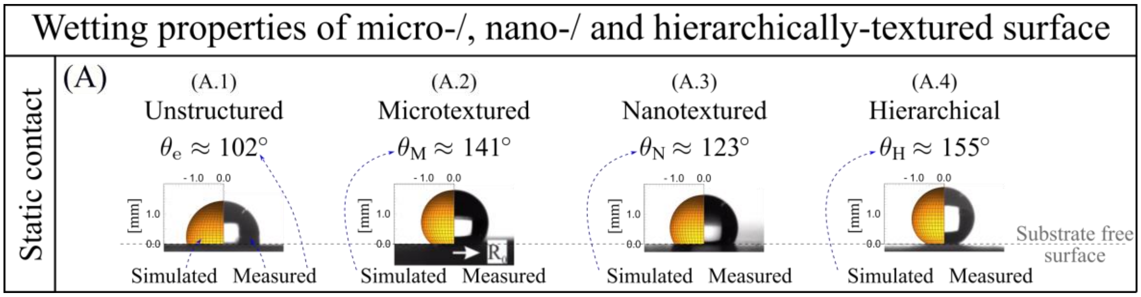

Experimental and simulation results of macroscopic static wetting properties (sessile droplet). (A) Measured and simulated droplet contact for the untextured (flat, A.1), microtextured (A.2), nanotextured (A.3) and hierarchical (A.4) surfaces.

Fig.SS6.

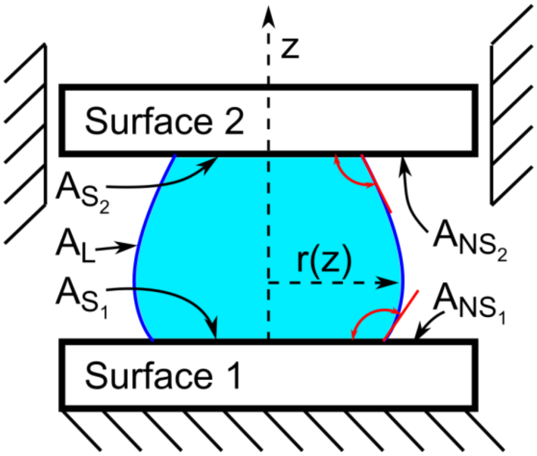

Schematic of the model describing a droplet squeezed among two dissimilar nominally flat surfaces.

Fig.SS7.

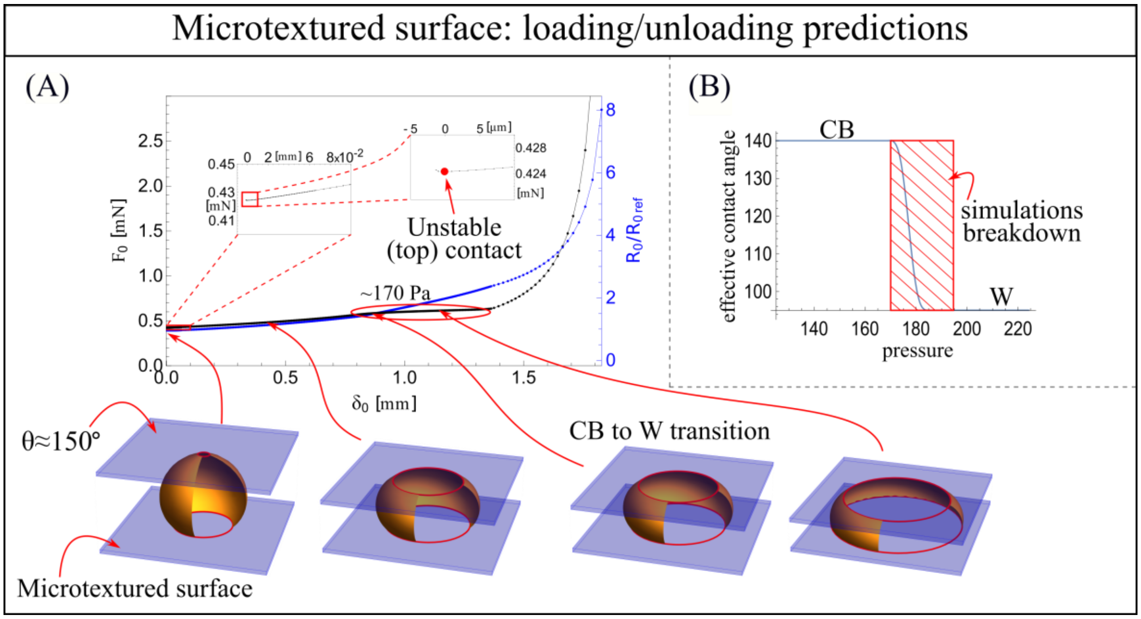

Simulation results: Force-displacement for the case where the droplet is squeezed between the microtextured surface (bottom plate) and a hydrophobic surface (top plate, see Fig.SS10). (A) Force (black line) and relative contact radius (blue line), predicted on the bottom plate, as a function of the top plate relative displacement. (B) Adopted relationship between apparent contact angle on the microstructure and pressure.

Fig.SS8.

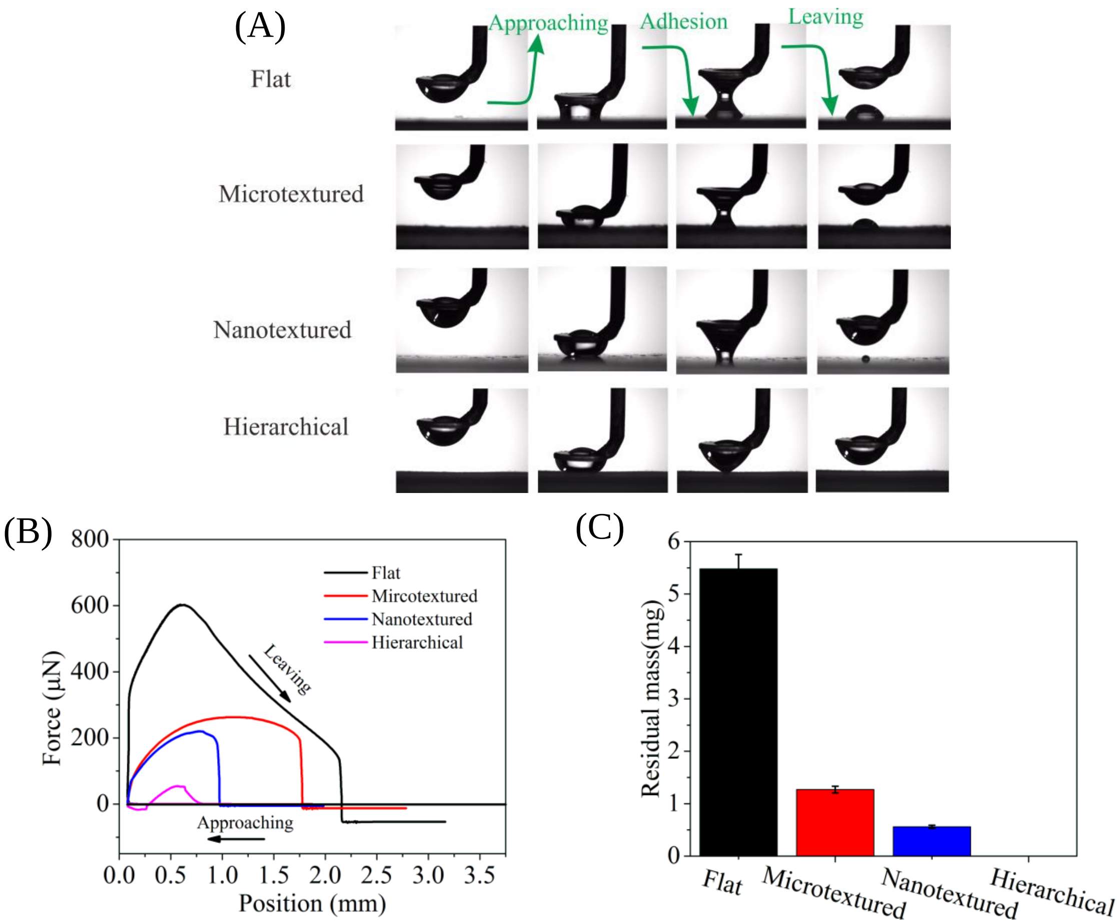

Wetting behaviour of the PP triboelectric layer when a water droplet is pressed against the different surfaces (flat, microtextured, nanotextured, hierarchical). (A) Photographic sequence (using a CCD camera) showing the loading and unloading stages of the adherence measuring process for the four different surfaces. (B) Force-distance curves of the water droplet (15 μL) approaching, contacting, leaving, and separating process with the PP triboelectric layer. (c) Residual mass of the water droplet after measurement.

Fig.SS9.

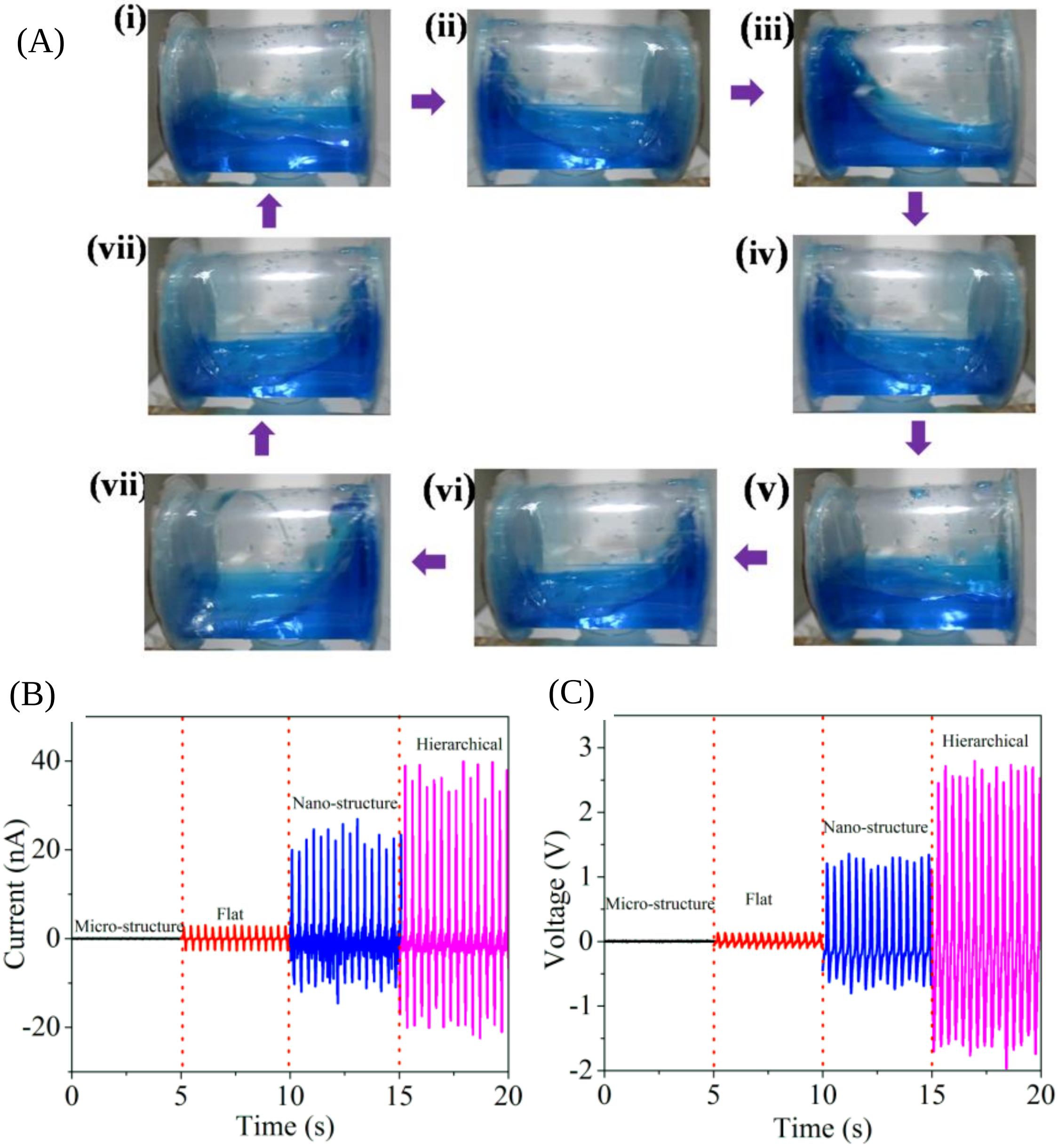

(A) Typical sloshing cycle adopted for the triboelectrical power generation of the water-based TENG, with water-to-cylinder volume ratio 0.3, vibration frequency 3 Hz, and vibration amplitude 5 mm. (B) and (C) Experimental results showing the steady-state triboelectrical outputs generated from the water-based TENG.

Fig.SS10.

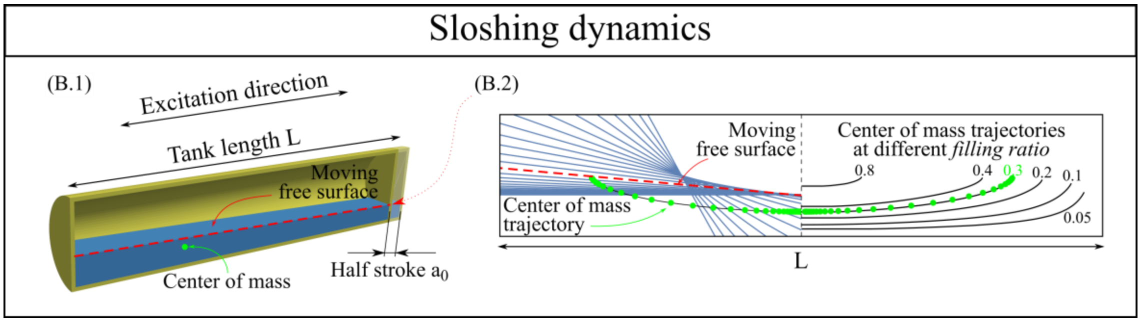

(A.1) Schematics of the water-based TENG, with indication of the excitation direction and parameters. (A.2) Left: Cross section of the tank showing the water free surface (red dashed line) and center of mass (green circle) trajectory during the sloshing. Right: center of mass trajectories at varying tank filling ratio. The green curve is for the sloshing condition which provides nearly the highest sloshing pressures, adopted through the following simulations and experiments.

Fig.SS11.

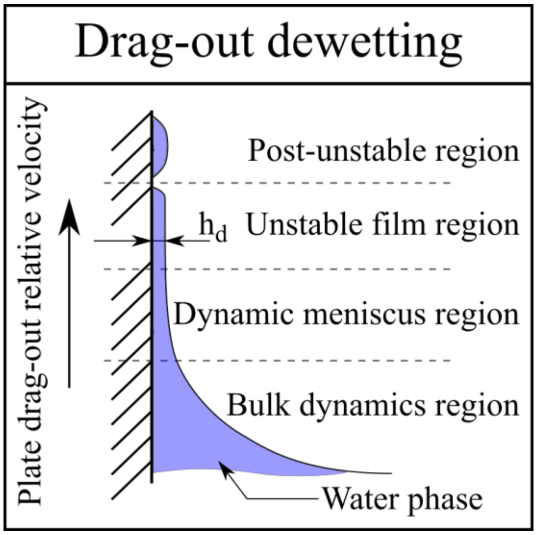

Schematics showing the fluid dynamics mechanisms involved in the drag-out dewetting process, with indication of the bulk region, the dynamic meniscus region, the unstable film region (if any), and the post-unstable region (if any). The existence of unstable and post-unstable ranges depends on the availability of a spontaneous film breakdown and external force-induced dewetting.

Fig.SS12.

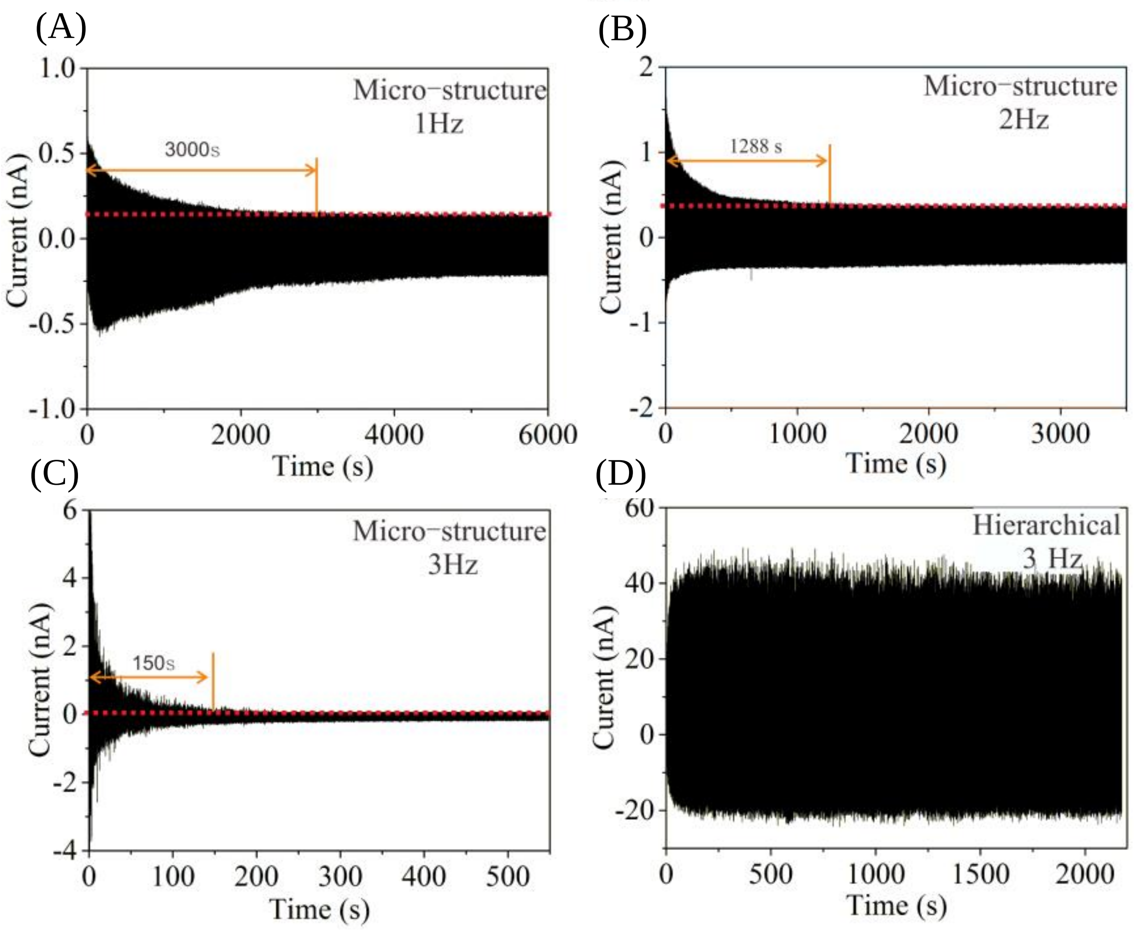

Measured transient output current for the TENG with microtextured PP surface at different frequency: (A) 1 Hz, (B) 2 Hz, (C) 3 Hz. (D) Measured transient output currents for the TENG with hierarchically structured PP surface at 3Hz.

Fig.SS13.

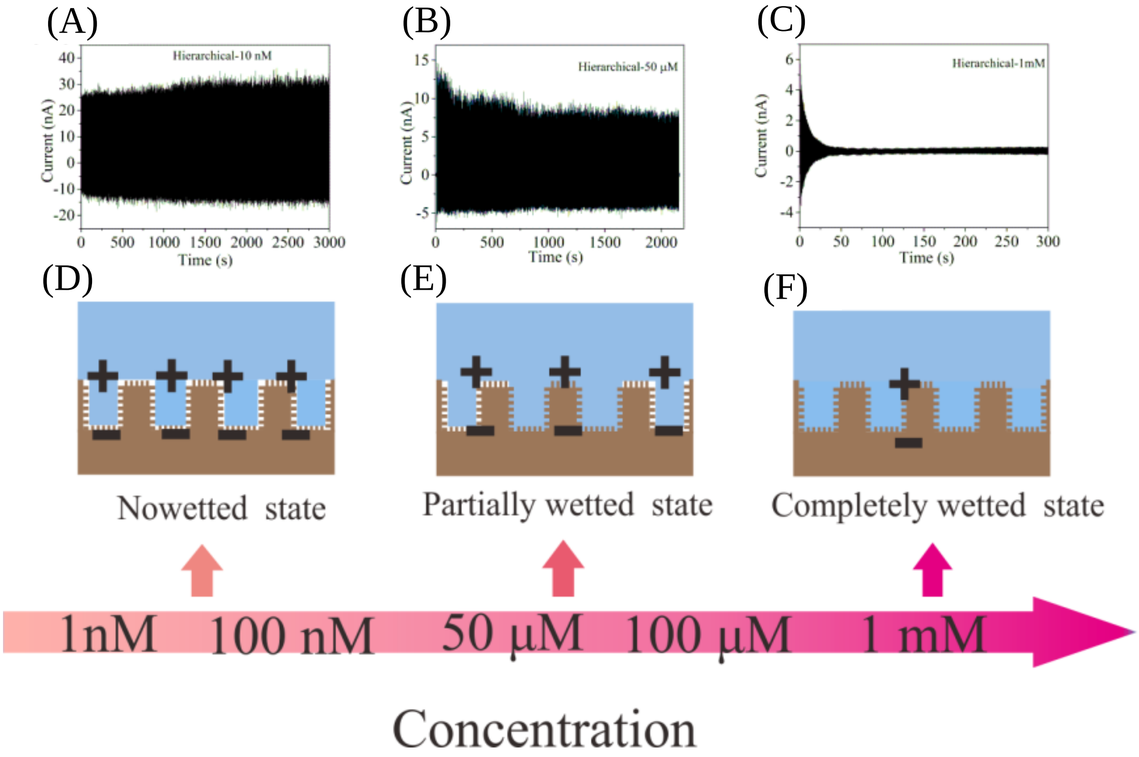

Full Wenzel transition with the increase of the concentration of surfactant. Measured transient tribocurrent of the water-based hierarchical TENG as a function of time for different surfactant concentration: (A) 10 nM, (B) 50 μM, and (C) 1 mM. Schematics of the wetting transitions occurring during surfactant injection: (D) nowetted state, (E) partially wetted state, and (F) full wetted state.

Fig.SS14.

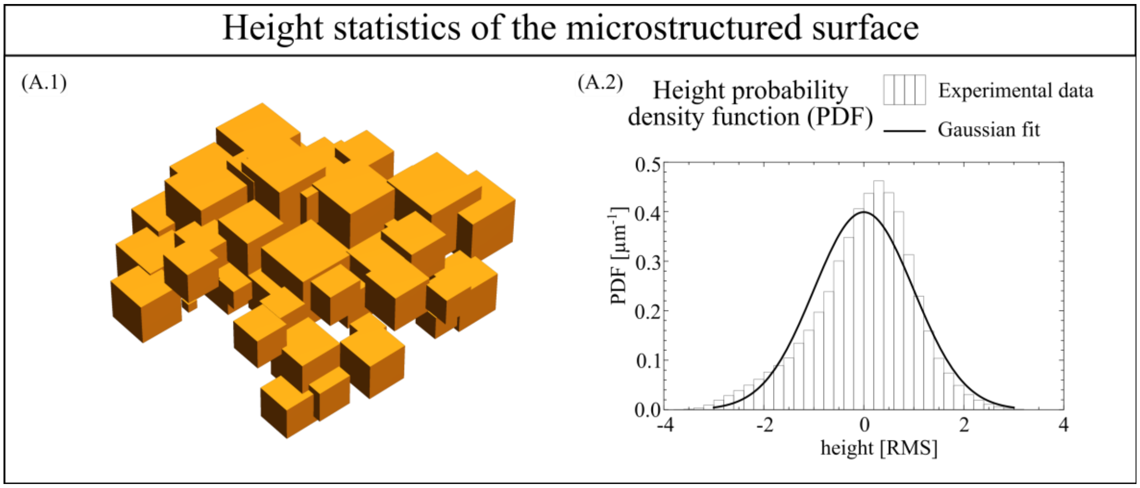

(A.1) Subset of microcubes of a generic microtextured virtual prototype. (A.2) Height probability density function of the microtextured surface roughness, skewed by the microcube geometries.

Fig.SS15.

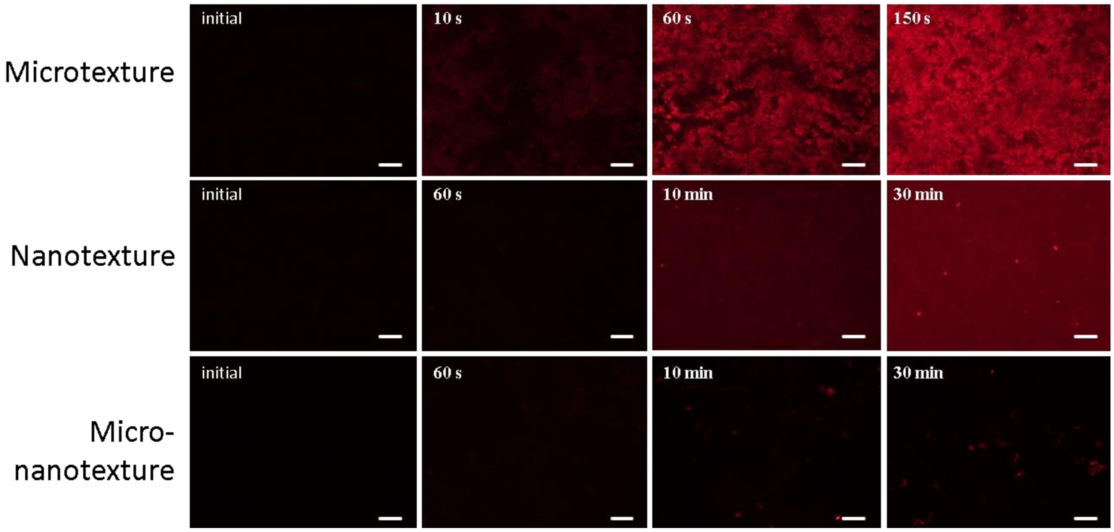

Epifluorence imaging of the residual wetting (irreversibly infiltrated fluid) on the textured PP surfaces at different sloshing times (3Hz sloshing frequency). RodhamineB is dissolved in water with concentration 0.01mg/ml. The bar corresponds to 200 µm.

Fig.SS16.

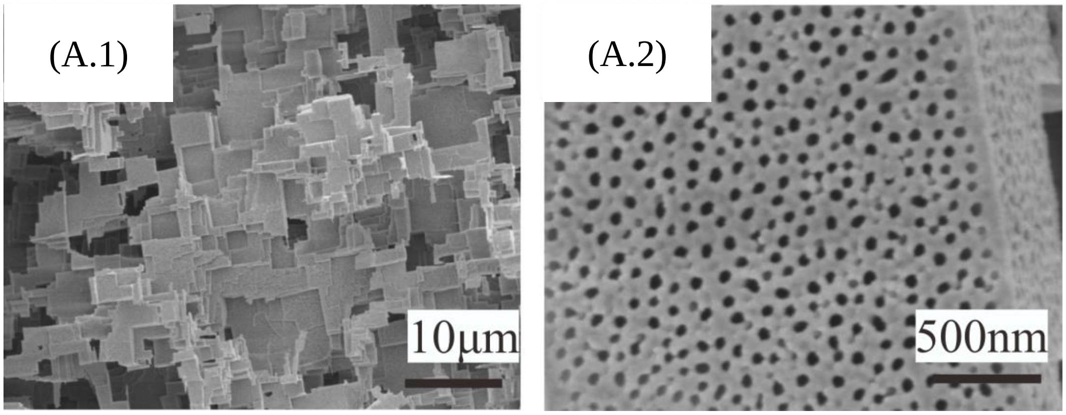

FESEM images of the hierarchical AAO templates with the low (A.1) and high (A.2) magnifications.

SI References

1. M. Cheng, *et al.*, Surface adhesive forces: a metric describing the drag-reducing effects of superhydrophobic coatings. *Small* **11**(14), 1665-1671 (2015).
2. H. Zou, *et al.*, Quantifying the triboelectric series. *Nat. Commun.* **10**(1), 1427 (2019).
3. D. Choi, *et al.*, Spontaneous electrical charging of droplets by conventional pipetting. *Sci. Rep.* **3**, 2037 (2013).
4. X. Zhang, *et al.*, Solid-liquid triboelectrification in smart U-tube for multifunctional sensors. *Nano Energy* **40**, 95-106 (2017).
5. R. A. Ibrahim, *et al.*, Recent advances in liquid sloshing dynamics. *Appl. Mech. Rev.* **54**(2), 133-199 (2001).
6. E. Rolley, C. Guthmann, R. Gombrowicz, V. Repain, Roughness of the Contact Line on a Disordered Substrate. *Phys. Rev. Lett.* **80**(13), 2865 (1998).
7. P. Volodin, A. Kondyurin, Dewetting of thin polymer film on rough substrate: I. Theory. *J. Phys. D: Appl. Phys.* **41**, 065306 (2008).
